# Perceived need-supportive biology course climate, learning engagement, and continuing biology learning intention: a cross-sectional SEM study of Chinese university students

**DOI:** 10.3389/fpsyg.2026.1913690

**Published:** 2026-08-26

**Authors:** Danni Mou, Chenglong Wang, Jun Jiang

**Affiliations:** School of Education Science, Harbin Normal University, Harbin, China

**Keywords:** basic psychological needs, biology education, continuing learning intention, learning engagement, need-supportive course climate, science identity, self-determination theory, self-efficacy

## Abstract

Grounded in self-determination theory, this study examined how Chinese university students’ perceived need-supportive biology course climate was associated with basic psychological needs, biology learning self-efficacy, science identity, biology learning engagement, and continuing biology learning intention. A total of 624 students from five anonymized Chinese higher education institutions completed an anonymous cross-sectional questionnaire. Confirmatory factor analysis, structural equation modeling, bootstrapped indirect-association analysis with 5,000 resamples, and multi-group SEM were conducted. The preferred correlated first-order measurement model yielded χ^2^ = 2,193.75, df = 1,139, CFI = 0.941, TLI = 0.934, RMSEA = 0.039, and SRMR = 0.045. The structural model yielded χ^2^ = 2,354.62, df = 1,166, CFI = 0.935, TLI = 0.929, RMSEA = 0.040, and SRMR = 0.048. Thus, CFI and TLI remained below the more stringent 0.95 reference value, whereas RMSEA and SRMR indicated comparatively favorable fit. Perceived need-supportive course climate was positively associated with basic psychological needs, *β* = 0.553, *p* < 0.001. Basic psychological needs were positively associated with biology learning self-efficacy, *β* = 0.310, *p* < 0.001, science identity, *β* = 0.286, *p* < 0.001, and learning engagement, *β* = 0.274, *p* < 0.001. Learning engagement was positively associated with continuing biology learning intention, *β* = 0.248, *p* < 0.001. The direct association between perceived course climate and continuing intention was not statistically clear, *β* = 0.047, *p* = 0.243, whereas several bootstrapped indirect associations were supported. Multi-group analyses showed only small changes in CFI and RMSEA across increasingly constrained models, supporting cautious comparison across the participating groups. Overall, the findings provide cross-sectional evidence that perceived biology course climate is associated with continuing biology learning intention through several motivational and engagement-related pathways. Longitudinal and intervention studies are needed to examine the temporal and causal ordering of these associations.

## 1 Introduction

Biology courses in university are an important part of science education as they familiarize students with the ideas, approaches, and modes of thought that can be applied to life sciences, health sciences, environmental problems, and scientific literacy in general. To most students, nevertheless, learning biology does not depend on the process of acquiring the information alone. It is about whether they find themselves able to learn biology, whether the science is personally relevant, whether they participate behaviorally and mentally in class activities, and whether they plan to keep learning biology-related knowledge. As indicated by previous studies on introductory STEM classes, student engagement is influenced by personal beliefs and also the learning environment, particularly in classes that students might think of as being demanding, competitive, or unrelated to their own objectives (Gasiewski et al., 2012; Sinatra et al., 2015).

The concept of student engagement has frequently been seen as a multidimensional concept with behavioral, emotional, and cognitive components (Fredricks et al., 2004). The multidimensional perspective is particularly applicable to science learning where engagement might involve long-term involvement in course-related activity, conceptual exertion, emotional curiosity, and eagerness to tackle uncertainty or challenge (Sinatra et al., 2015). Engagement is also closely linked to students’ persistence in their studies and future educational decisions in higher education. Engaged students in science learning may be more inclined to retain their interest in STEM-related disciplines and further develop scientific competencies (Maltese and Tai, 2011; Perez et al., 2014).

The theory of self-determination is a valuable model to describe the reasons why students’ perceptions of the course environment can be linked to their learning results. Students are more prone to foster autonomous motivation, adaptive engagement, and positive learning experience when their fundamental psychological needs of autonomy, competence, and relatedness are both encouraged and fulfilled (Deci and Ryan, 2000; Ryan and Deci, 2000, 2017). Autonomy support (autonomy), structure or competence support (structure), and interpersonal relatedness are frequently thought of as major aspects in need-supportive learning environments in educational contexts (Niemiec and Ryan, 2009; Jang et al., 2010; Reeve, 2012). A biology class climate where students get an opportunity to comprehend the worth of the given learning tasks, experience advancement, obtain constructive assistance, and sense teacher and peer respect could thus be indicative of enhanced motivational resources in regard to learning biology.

The available literature offers strong support for the applicability of self-determination theory to educational settings. Supportive teaching based on needs has been linked to student motivation, involvement, and adaptive classroom behavior (Skinner and Belmont, 1993; Reeve et al., 2004; Jang et al., 2010). Nevertheless, there are a number of gaps present. Firstly, most of the SDT-related educational studies have dealt with school or university learning in general, and fewer studies have focused on the learning of university biology as a particular discipline. The courses of biology demand the ability to interpret complex systems, use scientific evidence, and relate abstract facts to real-life events. These characteristics can be especially important when it comes to the perception of course climate by students. Secondly, studies in science education have tended to investigate constructs like science self-efficacy, science identity, and STEM persistence, but these constructs have not been frequently incorporated into a unified SDT-based framework connecting course climate, fundamental psychological needs, motivational beliefs, engagement, and future learning intention. Thirdly, there is little information on the comparability of such a model between various types of Chinese higher education institutions.

The current research fills these gaps through the examination of an integrated cross-sectional SEM model of Chinese university students who have taken biology-related courses. This model is associated with perceived need-supportive biology course climate and basic psychological needs, biology learning self-efficacy, science identity, and biology learning engagement and continuation of biology learning. In this article, the term “course climate” is used to describe student perception of autonomy support, competence support, and relatedness support in the context of biology learning. The term “continuing biology learning intention” means students report their intention to continue learning about the biology field or courses, which is regarded as a hypothetical future behavior. As the data are cross-sectional and self-reported, the research looks at associations and indirect relationships rather than causations.

The present research contributes to the literature in three ways. It is theoretical because it builds on the self-determination theory in university biology learning by examining a framework where fundamental psychological needs are considered as a middle-level motivational factor that connects perceived course climate with self-efficacy, science identity, engagement, and continuing intention. It empirically combines two concepts that have been extensively studied in science education, i.e., self-efficacy and science identity, into an SDT-based pathway model. In practice, it is the identification of characteristics of course climates that can be useful in promoting more involved biology learning experiences within Chinese higher education.

## 2 Theoretical background and research questions

### 2.1 Self-determination theory and need-supportive biology course climate

Self-determination theory proposes that human motivation and psychological functioning are partly shaped by the satisfaction of three basic psychological needs: autonomy, competence, and relatedness (Deci and Ryan, 2000; Ryan and Deci, 2000, 2017). Autonomy refers to experiencing one’s learning activities as volitional and personally meaningful, competence refers to feeling effective and capable of meeting learning challenges, and relatedness refers to experiencing connection, respect, and support from others. Satisfaction of these needs has been associated with adaptive motivation and engagement in educational settings (Vansteenkiste et al., 2020). A need-supportive learning environment provides students with meaningful choices and rationales, appropriately challenging tasks, constructive feedback, and interpersonal support (Niemiec and Ryan, 2009; Reeve, 2012). Autonomy support may involve explaining the value of learning activities, acknowledging students’ perspectives, and reducing unnecessary controlling pressure. Competence support may involve clear guidance, appropriate levels of challenge, constructive feedback, and opportunities to experience progress. Relatedness support may be reflected in respectful teacher–student relationships, supportive peer interaction, and an inclusive classroom climate. Earlier studies indicate that autonomy support and structure are not opposing teaching principles, but students are better off when they have access to both during learning activities (Jang et al., 2010).

The supportive climate in regard to needs can be particularly useful in a biology class, as students frequently have to harmonize factual information, conceptual thinking, laboratory or evidence-based thinking, and personal interpretations of the event that are meaningful to them. In cases when students feel that a biology course helps them to satisfy their autonomy, competence, and relatedness, they might tend to perceive their fundamental psychological needs as satisfied.

A need-supportive climate may be particularly relevant in biology courses because students are often required to integrate factual knowledge, conceptual reasoning, evidence-based thinking, and personally meaningful interpretations of biological phenomena. When students perceive that a biology course provides meaningful choice, clear support for competence, and respectful interpersonal relationships, they may be more likely to experience their needs for autonomy, competence, and relatedness as satisfied. Accordingly, perceived need-supportive biology course climate is expected to be positively associated with students’ basic psychological need satisfaction.

### 2.2 Basic psychological needs as proximal motivational experiences

Basic psychological need satisfaction can be regarded as a proximal motivational experience linking educational environments with learning-related outcomes. Previous classroom research has shown that teacher behavior and instructional support are associated with student engagement, partly because they shape students’ perceptions of autonomy, competence, and interpersonal connection (Skinner and Belmont, 1993; Reeve et al., 2004; Jang et al., 2010). From an SDT perspective, course environments are not expected to influence engagement solely through external demands or performance pressures. Rather, their motivational relevance depends partly on whether students experience themselves as autonomous, capable, and socially connected learners.

In the present framework, basic psychological need satisfaction is positioned as a proximal motivational experience linking perceived course climate with subsequent learning-related outcomes. This position is consistent with SDT, which proposes that social-contextual support becomes motivationally relevant partly through the satisfaction of students’ psychological needs (Deci and Ryan, 2000; Vansteenkiste et al., 2020). In biology learning, students whose needs are more strongly satisfied may be more likely to view themselves as capable of mastering biology-related tasks and to connect biology learning with their developing science-related self-concept. Accordingly, greater basic psychological need satisfaction is expected to be associated with stronger biology learning self-efficacy and science identity.

### 2.3 Biology learning self-efficacy and science identity

The concept of self-efficacy is the belief of people about their ability to plan and perform activities necessary to achieve desired results (Bandura, 1977, 1997). Self-efficacy has been linked to both persistence and effort, as well as strategy use and behaviors related to achievement in academic contexts (Pajares, 1996; Schunk and Pajares, 2002). The concept of science self-efficacy is especially significant in the context of science learning since students are frequently faced with such complex ideas, unclear task-solving problems, and discipline practices that demand prolonged efforts (Britner and Pajares, 2006). Biology learning self-efficacy in this research study means that the student feels that they have the capacity to learn biology content, do biology learning tasks, and overcome difficulties in biology courses.

The concept of science identity is theoretically different than self-efficacy. Self-efficacy is a measure of perceived capability, whereas science identity is a measure of how much students view science as part of their identity and feel recognized as a science-related learner or person (Carlone and Johnson, 2007; Hazari et al., 2010). The concept of identity in the field of science education has been associated with student persistence, career-related decisions, and membership in science communities (Chemers et al., 2011; Stets et al., 2017). Life science education scholarship has likewise focused on the importance of self-efficacy, belonging, and science identity to student learning experiences (Trujillo and Tanner, 2014).

Both self-efficacy and science identity may play a role in engagement via various paths of motivation. Students who have higher self-efficacy in learning biology might be more inclined to put in the effort and apply cognitive strategies due to their belief in their ability to succeed. Those students who have higher science identity may have greater emotional and personal investment as the process of learning biology is linked to the way they perceive themselves and their future opportunities. Earlier studies imply that self-efficacy and identity can both be important factors in science commitment and STEM persistence, but they cannot be viewed as identical constructs (Chemers et al., 2011; Robnett et al., 2015). Hence, the current model consists of both constructs as predictors of biology learning engagement.

Biology learning self-efficacy and science identity may contribute to engagement through related but conceptually distinct motivational processes. Students with stronger biology learning self-efficacy may be more willing to invest effort and use cognitive strategies because they believe that they can successfully meet learning demands. Students with a stronger science identity may show greater emotional and personal investment because biology learning is more closely connected with how they understand themselves and their future possibilities. Previous research suggests that self-efficacy and identity are both relevant to science commitment and STEM persistence, although they represent distinct constructs (Chemers et al., 2011; Robnett et al., 2015). Accordingly, both biology learning self-efficacy and science identity are included in the proposed model as theoretically distinct correlates of biology learning engagement.

### 2.4 Biology learning engagement and continuing biology learning intention

The concept of learning engagement can be described as a multifaceted element that consists of behavioral participation, emotional involvement, and cognitive investment (Fredricks et al., 2004; Appleton et al., 2006). Multidimensionality means that it is significant when talking about science education since students can engage in visible activities in classes but differ in conceptual efforts, emotional interests, and willingness to apply scientific reasoning (Sinatra et al., 2015). Engagement can also be considered as one of the main motivational results of SDT because it shows that students are actively engaged in the learning process as opposed to simply complying with course demands (Reeve, 2012).

Continuing biology learning intention is treated in this study as a distal self-reported learning intention. It refers to students’ willingness to continue learning biology-related content, take further biology-related courses, or maintain an interest in biology learning. This construct is related to, but distinct from, actual persistence or subsequent course enrollment. Previous STEM research has shown that students’ beliefs, values, identities, and learning experiences are associated with persistence and future participation in science-related pathways (Tai et al., 2006; Maltese and Tai, 2011; Perez et al., 2014). Within the present framework, biology learning engagement is expected to be positively associated with continuing biology learning intention because students who are more behaviorally, cognitively, and emotionally engaged may also report stronger willingness to continue learning biology.

Although engagement is expected to be a proximal predictor of continuing intention, the model also allows basic psychological needs, self-efficacy, and science identity to have direct associations with continuing intention. This is consistent with prior research showing that science self-efficacy and science identity are related to science commitment and persistence-related outcomes (Chemers et al., 2011; Perez et al., 2014; Stets et al., 2017). However, because this study is cross-sectional, these paths are interpreted as associations rather than developmental or causal sequences.

### 2.5 Indirect pathways from course climate to engagement and continuing intention

A key assumption of the present study is that perceived need-supportive biology course climate may be linked to engagement and continuing intention through a chain of motivational and identity-related variables. SDT suggests that need-supportive environments foster adaptive learning outcomes by supporting students’ basic psychological needs (Deci and Ryan, 2000; Vansteenkiste et al., 2020). Science education research further suggests that self-efficacy and identity are relevant for students’ persistence and science-related commitment (Carlone and Johnson, 2007; Chemers et al., 2011; Robnett et al., 2015). Taken together, these perspectives suggest that course climate may not be associated with continuing biology learning intention only through a direct path. Instead, its association may be carried through students’ need satisfaction, capability beliefs, science-related self-concept, and engagement.

Accordingly, the present study examines several theoretically specified indirect associations. First, perceived need-supportive biology course climate is examined in relation to biology learning engagement through basic psychological needs. Second, an indirect association through basic psychological needs and biological learning self-efficacy is considered. Third, a parallel pathway through basic psychological needs and science identity is examined. Finally, the model examines whether perceived course climate is indirectly associated with continuing biology learning intention through motivational and engagement-related pathways. Given the cross-sectional design, these pathways are evaluated as statistical indirect associations rather than as evidence of causal mediation. These direct and indirect relationships are addressed collectively in RQ2.

### 2.6 Institution type as a contextual comparison

The institutions involved were of various types of higher education settings within China, such as comprehensive education, teacher training, vocational undergraduate training, and agricultural/life-science institutions. The type of institution might be indicative of how courses are positioned, the makeup of students, the discipline culture, and the perceived value of learning biology. Previous studies about the involvement in STEM have highlighted the fact that the learning experience of students is influenced by various contextual factors such as the organization of a course, institutional context, and peer community (Gasiewski et al., 2012; Wilson et al., 2015). Hence, it is useful to check if the suggested model can be compared between institution types.

In the present study, institution type is treated as a contextual grouping variable rather than as a causal predictor. The analyses first assess whether the measurement structure is broadly comparable across the participating groups and then explore whether the principal structural associations show similar patterns across groups. Because the group sizes are unequal and these comparisons are exploratory, group-specific estimates are interpreted cautiously rather than as evidence of general institutional-type differences.

### 2.7 Research questions and the present study

Based on the theoretical framework described above, the present study addressed three research questions:

*RQ1*: Does the proposed measurement model provide adequate reliability and construct-validity evidence for the focal constructs among Chinese university students?

*RQ2*: What direct and indirect statistical associations are observed among perceived need-supportive biology course climate, basic psychological needs, biology learning self-efficacy, science identity, biology learning engagement, and continuing biology learning intention?

*RQ3*: To what extent are the measurement properties and structural associations broadly comparable across the participating institution-type groups?

**Figure 1:**
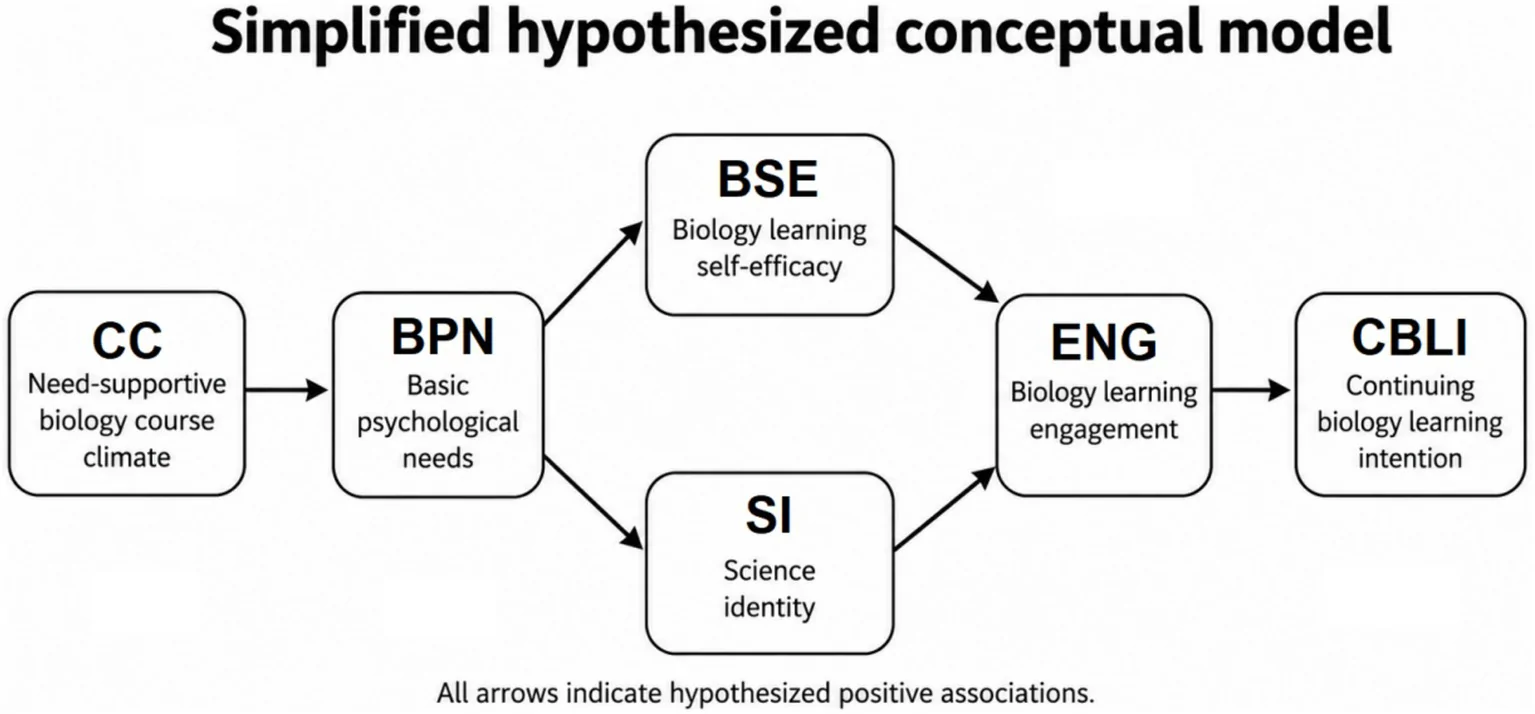
Proposed SDT-based structural model of biology learning engagement and continuing biology learning intention. CC, perceived need-supportive biology course climate; BPN, basic psychological needs; BSE, biology learning self-efficacy; SI, science identity; ENG, biology learning engagement; CBLI, continuing biology learning intention. Solid arrows indicate theoretically specified statistical associations. Additional direct paths to CBLI were estimated in the structural model but are not displayed for visual clarity. Institution type was examined as a contextual grouping variable and is not displayed in the primary theoretical model. Because the study used cross-sectional self-report data, the arrows represent statistical associations rather than causal effects.

To address these questions, the study tested a cross-sectional SDT-based structural model linking perceived need-supportive biology course climate with basic psychological needs, biology learning self-efficacy, science identity, biology learning engagement, and continuing biology learning intention. Figure 1 presents the proposed model. The model was used to examine both the primary structural associations and the theoretically specified indirect associations, together with exploratory comparisons across the participating institutional groups. Because the data are cross-sectional and self-reported, all structural paths and indirect pathways are interpreted as statistical associations rather than as evidence of temporal or causal ordering.

## 3 Materials and methods

### 3.1 Study design

This study used a cross-sectional survey design to examine the associations among perceived need-supportive biology course climate, basic psychological needs, biology learning self-efficacy, science identity, biology learning engagement, and continuing biology learning intention among Chinese university students. The study was non-experimental and anonymous. No course intervention, pre-test/post-test design, teacher-level data, class-level data, attendance records, course scores, teacher interviews, or student reflections were collected.

The design was selected because the aim was to test a theory-based structural model rather than evaluate a specific teaching intervention. Accordingly, all paths in the model were interpreted as cross-sectional associations. The terms “direct effect,” “indirect effect,” and “pathway” are used in their statistical SEM sense and do not imply causal inference.

### 3.2 Participants and setting

The analytic sample was a group of six hundred and twenty-four university students in five unspecified higher education institutions in China. They were named as Institution A, Institution B, Institution C, Institution D, and Institution E. As a measure of confidentiality of institutions, no actual institution names are given in the dataset. Four institution types were represented by the five institutions: comprehensive university, teacher education university, applied undergraduate university, and agricultural/life-science university.

Among the 624 participants, 406 were female (65.1%), and 218 were male (34.9%). To reduce potential identifiability, age was recorded in categories rather than as an exact age. A total of 327 participants were 18–20 years old (52.4%), 254 were 21–23 years old (40.7%), and 43 were 24 years or older (6.9%). Most participants were undergraduates (*n* = 556, 89.1%), while 68 were postgraduate students (10.9%). The sample included students from different stages of university study, ranging from first-year undergraduates to third-year postgraduate students.

Students’ academic backgrounds were diverse. Biological sciences represented the largest major category (*n* = 182, 29.2%), followed by other STEM fields (*n* = 94, 15.1%), agriculture/life sciences (*n* = 78, 12.5%), education (*n* = 73, 11.7%), environmental science (*n* = 62, 9.9%), medicine/health sciences (*n* = 45, 7.2%), humanities/social sciences (*n* = 43, 6.9%), business/management (*n* = 31, 5.0%), and arts and design (*n* = 16, 2.6%). A total of 405 students (64.9%) reported that their major was biology-related, whereas 219 students (35.1%) reported that their major was not biology-related.

Participants were enrolled in different types of biology-related courses or modules. These included major required biology courses (*n* = 198, 31.7%), laboratory-supported biology courses (*n* = 117, 18.8%), elective biology modules (*n* = 99, 15.9%), general education biology courses (*n* = 118, 18.9%), and life science literacy courses (*n* = 92, 14.7%). Students also reported different levels of prior biology background: limited (*n* = 121, 19.4%), moderate (*n* = 331, 53.0%), and advanced (*n* = 172, 27.6%). Biology course interest was reported as low by 97 students (15.5%), moderate by 342 students (54.8%), and high by 185 students (29.6%). Participant characteristics are reported in Table 1.

**Table 1:** Participant characteristics of the analytic sample.

Variable	Category	*n*	%
Total	All participants	624	100.0
Institution code	Institution A	132	21.2
Institution code	Institution B	118	18.9
Institution code	Institution C	114	18.3
Institution code	Institution D	145	23.2
Institution code	Institution E	115	18.4
Institution type	Comprehensive university	247	39.6
Institution type	Teacher education university	118	18.9
Institution type	Applied undergraduate university	114	18.3
Institution type	Agricultural/life-science university	145	23.2
Gender	Female	406	65.1
Gender	Male	218	34.9
Age group	18–20	327	52.4
Age group	21–23	254	40.7
Age group	24 and above	43	6.9
Education level	Undergraduate	556	89.1
Education level	Postgraduate	68	10.9
Year of study	Year 1	152	24.4
Year of study	Year 2	177	28.4
Year of study	Year 3	139	22.3
Year of study	Year 4	88	14.1
Year of study	Postgraduate year 1	30	4.8
Year of study	Postgraduate year 2	21	3.4
Year of study	Postgraduate year 3	17	2.7
Major category	Biological sciences	182	29.2
Major category	Agriculture/life sciences	78	12.5
Major category	Medicine/health sciences	45	7.2
Major category	Environmental science	62	9.9
Major category	Education	73	11.7
Major category	Other STEM	94	15.1
Major category	Humanities/social sciences	43	6.9
Major category	Business/management	31	5.0
Major category	Arts and design	16	2.6
Biology-related major	Yes	405	64.9
Biology-related major	No	219	35.1
Course type	Major required biology course	198	31.7
Course type	Laboratory-supported biology course	117	18.8
Course type	Elective biology module	99	15.9
Course type	General education biology course	118	18.9
Course type	Life science literacy course	92	14.7
Prior biology background	Limited	121	19.4
Prior biology background	Moderate	331	53.0
Prior biology background	Advanced	172	27.6
Biology course interest	Low	97	15.5
Biology course interest	Moderate	342	54.8
Biology course interest	High	185	29.6

*N* = 624. Institution names were anonymized as Institution A–E. Percentages may not sum to 100 because of rounding.

### 3.3 Procedure and ethical considerations

Anonymous questionnaires were used to gather data on students’ experiences in learning biology. The survey gathered demographic data and self-reported answers to the central constructs of the study. It was voluntary. Names, student numbers, contact details, class ID, teacher ID or any other personal identification number were not recorded in the questionnaire. Before analysis, institution names were substituted with anonymous institution codes.

This was a non-experimental educational survey that was anonymous and had low risk. There was no clinical, psychological diagnostic, or sensitive health data collected. The minors were not included since the youngest age group recorded was 18 years. Completion of the questionnaire was considered as informed consent to be part of the research.

Anonymized data were used exclusively as research materials. Since the study was based on self-report information, the responses of the participants were based on how they perceived their learning experiences instead of how they were observed by others in the classroom or their academic performance measured objectively.

### 3.4 Instrument

The questionnaire included background variables and 50 substantive Likert-type items. All substantive items were rated on a five-point scale ranging from 1 = strongly disagree to 5 = strongly agree. Higher scores indicated higher levels of the corresponding construct. The questionnaire was administered in Chinese. English item wordings are reported in Supplementary Appendix 1 for manuscript presentation.

The item pool was constructed to represent established constructs in self-determination theory and science education research. Items were organized according to the theoretical facets of each construct (Table 2). Scale scores were computed as the mean of their corresponding items. No reverse-coded items were used.

**Table 2:** Constructs, facets, item numbers, and example items.

Construct	Facet	Item codes	Items	Example item
Need-supportive biology course climate	Autonomy support	AS1–AS4	4	The biology course gives me opportunities to make meaningful choices in learning activities.
Need-supportive biology course climate	Competence support	CS1–CS4	4	The course provides clear guidance that helps me understand how to improve in biology.
Need-supportive biology course climate	Relatedness support	RS1–RS4	4	I feel respected when participating in biology learning activities.
Basic psychological needs	Autonomy need satisfaction	AUT1–AUT4	4	I feel that I have a sense of choice when learning biology.
Basic psychological needs	Competence need satisfaction	COM1–COM4	4	I feel capable of understanding important biology concepts.
Basic psychological needs	Relatedness need satisfaction	REL1–REL4	4	I feel connected with others during biology learning.
Biology learning self-efficacy	Biology learning self-efficacy	BSE1–BSE5	5	I am confident that I can understand core concepts in biology.
Science identity	Science identity	SI1–SI5	5	Biology learning helps me feel connected to scientific ways of thinking.
Biology learning engagement	Behavioral engagement	BE1–BE4	4	I participate actively in biology learning activities.
Biology learning engagement	Cognitive engagement	CEG1–CEG4	4	I try to connect new biology knowledge with what I have learned before.
Biology learning engagement	Emotional engagement	EE1–EE4	4	I feel interested when learning biology.
Continuing biology learning intention	Continuing biology learning intention	CBLI1–CBLI4	4	I intend to continue learning biology-related knowledge in the future.

All substantive items were rated from 1 = strongly disagree to 5 = strongly agree. AS, autonomy support; CS, competence support; RS, relatedness support; AUT, autonomy need satisfaction; COM, competence need satisfaction; REL, relatedness need satisfaction; BSE, biology learning self-efficacy; SI, science identity; BE, behavioral engagement; CEG, cognitive engagement; EE, emotional engagement; CBLI, continuing biology learning intention.

### 3.5 Measures

#### 3.5.1 Perceived need-supportive biology course climate

Perceived need-supportive biology course climate was measured with 12 items covering three facets: autonomy support, competence support, and relatedness support. The three-facet structure followed self-determination theory’s distinction between autonomy, competence, and relatedness support in educational settings (Deci and Ryan, 2000; Niemiec and Ryan, 2009; Reeve, 2012).

Autonomy support was measured with four items. A sample item was: “The biology course gives me opportunities to make meaningful choices in learning activities.” Competence support was measured with four items. A sample item was: “The course provides clear guidance that helps me understand how to improve in biology.” Relatedness support was measured with four items. A sample item was: “I feel respected when participating in biology learning activities.”

The total score for perceived need-supportive biology course climate was calculated as the mean of all 12 items, with higher scores indicating stronger perceived autonomy, competence, and relatedness support in the biology course climate.

#### 3.5.2 Basic psychological needs

The basic psychological needs satisfaction was assessed using 12 items, which represented the autonomy need satisfaction, competence need satisfaction, and relatedness need satisfaction. The structure was founded on self-determination theory that posits autonomy, competence, and relatedness as fundamental psychological needs of motivation and adaptive behavior (Deci and Ryan, 2000; Ryan and Deci, 2000; Vansteenkiste et al., 2020).

Autonomy need measures were assessed using four items. One example of an item is this: I believe that I have a feeling of choice in studying biology. Competence needs measures were assessed using four items. One example of an item is this: I feel competent to comprehend significant biology concepts. Relatedness need measures were assessed using four items. One example of an item is this: I feel linked to other people in the process of learning biology.

The overall score of the basic psychological needs was obtained through the mean of all 12 items. The high score signified higher levels of satisfaction of autonomy, competence, and relatedness needs in the process of learning biology.

#### 3.5.3 Biology learning self-efficacy

Self-efficacy of studying biology was assessed with the help of five items. The items were based on the perception of students that they can comprehend the concept of biology, apply their knowledge of biology, solve difficult problems, make use of the evidence, and enhance their learning methods. This construct was theoretically based on the self-efficacy theory and literature of academic self-efficacy (Bandura, 1977, 1997; Pajares, 1996; Britner and Pajares, 2006).

An example of an item was: “I will be sure to comprehend fundamental principles in the field of biology.” The scale score was determined by averaging the 5 items. The higher scores showed more perceived capability in learning biology.

#### 3.5.4 Science identity

Five items were used to measure science identity. The items measured the self-perception of students as learners in relation to science, their relationship to scientific thought processes, the level of recognition that students believed they received from significant others, and the importance of their biology studies in their academic identity. The construct was developed according to the science identity literature in science and STEM education (Carlone and Johnson, 2007; Hazari et al., 2010; Trujillo and Tanner, 2014).

One of the sample items was: Biology learning makes me feel that I belong to scientific thought patterns. This scale score was determined as the mean of five items. A high score meant a high strength of science-related identity in the context of biology learning.

#### 3.5.5 Biology learning engagement

The engagement of learning biology was evaluated using 12 items, including behavioral engagement, cognitive engagement, and emotional engagement. The multidimensional structure was based on previous engagement studies that define engagement as behavioral participation, cognitive investment, and emotional involvement (Fredricks et al., 2004; Appleton et al., 2006; Reeve, 2012; Sinatra et al., 2015).

The measure of behavioral engagement was four items. The example of an item is as follows: I actively engage in learning biology activities. Four items were used to measure cognitive engagement. An example of an item is as follows: I attempt to relate new information about biology to what I already know. Four items were used to assess emotional engagement. The example of an item is as follows: When studying biology, I am interested.

The mean of the total biology learning engagement score was obtained by calculating the average of all 12 items. The higher the scores were, the more intense the behavioral, cognitive, and emotional engagement in biology learning.

#### 3.5.6 Continuing biology learning intention

The measure of the intention to continue learning biology comprised four items. The questions used measured the willingness of students to keep on learning about biological concepts, enroll in more biology-related courses, apply the knowledge in their future education or other aspects of life, and follow the development of issues in the sphere of biology. The construct has been regarded as self-reported intention instead of actual future conduct. The decision to incorporate this result was grounded on the studies of STEM persistence and science learning continuation (Tai et al., 2006; Maltese and Tai, 2011; Perez et al., 2014).

An example item was: I plan to keep acquiring information about the field of biology in the future. The scale score was the mean of the four items. The high scores represented a stronger intention to continue learning biology.

#### 3.5.7 Background variables

The questionnaire also collected background variables, including institution code, institution type, gender, age group, education level, year of study, major category, whether the student’s major was biology-related, course type, prior biology background, and biology course interest. Institution type was used as the grouping variable in multi-group SEM. Other background variables were used to describe the sample.

### 3.6 Data screening and scoring

Data screening was conducted before the main analyses. Response ranges were checked to ensure that all substantive questionnaire responses were within the expected range from 1 to 5. The final analytic dataset contained complete responses for all analyzed items and constructs, so no missing-data imputation was applied.

Composite scale scores were computed by averaging the relevant items for each construct or facet. Scale-level descriptive statistics included the mean, standard deviation, median, minimum, maximum, skewness, kurtosis, and missing count. Item-level descriptive statistics were also examined, including item means, standard deviations, floor and ceiling percentages, corrected item-total correlations, and Cronbach’s alpha if an item was deleted.

Because all focal constructs were assessed by the same respondents at the same time point using a common self-report questionnaire and response format, common method variance was considered a potential source of bias. Procedurally, the anonymous survey format and the absence of personally identifiable information were intended to reduce evaluation concerns and socially desirable responding. However, these procedural features cannot eliminate method variance associated with common source, measurement occasion, or response format (Podsakoff et al., 2003, 2012). A one-factor CFA model was therefore included only as a limited diagnostic comparison with the theoretically specified measurement models. Poor fit of the one-factor model was interpreted as evidence that the observed item covariance could not be adequately represented by a single common factor; it was not interpreted as demonstrating the absence of common method variance.

### 3.7 Reliability and validity analysis

Internal consistency was assessed using Cronbach’s alpha and McDonald’s omega. Cronbach’s alpha was reported because it remains widely used in educational and psychological research, while McDonald’s omega was included as a complementary reliability estimate that does not rely on the same assumptions regarding tau-equivalence (McDonald, 1999; Dunn et al., 2014).

The composite reliability (CR) and average variance extracted (AVE) were used to determine convergent validity. The interpretation of the CR values was that they had to be 0.70 or higher to be acceptable. The AVE values, which were approximately 0.50, were interpreted carefully and combined with the loading of items and reliability evidence, in accordance with traditional practice in measurement of SEM-based measurements (Fornell and Larcker, 1981; Brown, 2015).

Confirmatory factor analysis was conducted to evaluate the measurement structure. Because the questionnaire items used ordered five-point Likert-type response categories, a WLSMV-compatible ordinal CFA approach was used. Four measurement models were compared. The first was a one-factor model in which all substantive items loaded on a single latent factor. The second was a six-factor construct-level model representing the six focal constructs: perceived need-supportive course climate, basic psychological needs, biology learning self-efficacy, science identity, biology learning engagement, and continuing biology learning intention. The third was a correlated first-order measurement model in which the theoretically specified first-order dimensions were modeled as correlated latent variables. The fourth was a higher-order measurement model in which multidimensional constructs were represented by their corresponding lower-order facets.

Model fit was evaluated using the chi-square statistic, degrees of freedom, comparative fit index (CFI), Tucker–Lewis index (TLI), root mean square error of approximation (RMSEA) with its 90% confidence interval, and standardized root mean square residual (SRMR). The fit indices were interpreted jointly rather than according to a single rigid cutoff. Values approaching 0.95 for CFI and TLI, approximately 0.06 or lower for RMSEA, and approximately 0.08 or lower for SRMR were treated as useful reference points for evaluating model fit (Hu and Bentler, 1999; Kline, 2016). Because these values are guidelines rather than universal decision rules, the magnitude and pattern of all indices were considered together when evaluating the measurement and structural models.

### 3.8 Correlation analysis

Pearson correlations were calculated among the six main scale scores: perceived need-supportive biology course climate, basic psychological needs, biology learning self-efficacy, science identity, biology learning engagement, and continuing biology learning intention. Two-tailed *p*-values were reported. The correlation analysis was used to provide an initial description of bivariate associations among the constructs before testing the structural equation model.

### 3.9 Structural equation modeling

Structural equation modeling was used to evaluate the proposed SDT-based model. The structural model retained the item-level measurement component and specified directional paths among the latent constructs according to the theoretical model. Perceived need-supportive biology course climate was modeled as the exogenous construct. Basic psychological needs, biology learning self-efficacy, science identity, and biology learning engagement were modeled as motivational and engagement-related mediating constructs. Continuing biology learning intention was modeled as the distal outcome.

The primary model estimated direct paths from perceived need-supportive course climate to basic psychological needs, biology learning self-efficacy, science identity, biology learning engagement, and continuing biology learning intention. Basic psychological needs were specified as predictors of biology learning self-efficacy, science identity, learning engagement, and continuing intention. Biology learning self-efficacy and science identity were specified as predictors of learning engagement and continuing intention. Biology learning engagement was specified as a predictor of continuing biology learning intention.

Standardized path coefficients, standard errors, z or t values, p-values, 95% confidence intervals, and R^2^ values were reported. Model fit was evaluated using the same global fit indices as in the CFA.

### 3.10 Indirect-association analysis

Indirect statistical associations were examined using bootstrapped confidence intervals. Bootstrapping was used because the sampling distributions of indirect associations may be non-normal, and resampling-based confidence intervals provide a direct means of evaluating whether an estimated indirect association differs from zero (Preacher and Hayes, 2008). A total of 5,000 bootstrap resamples were used. For each resample, cases were sampled with replacement, the specified structural model was re-estimated, and the corresponding indirect associations were calculated. An indirect association was considered statistically supported when its bootstrapped 95% confidence interval did not include zero.

The theoretically specified indirect pathways were: (1) perceived course climate → basic psychological needs → learning engagement; (2) perceived course climate → basic psychological needs → biology learning self-efficacy → learning engagement; (3) perceived course climate → basic psychological needs → science identity → learning engagement; and (4) perceived course climate → basic psychological needs → learning engagement → continuing biology learning intention. The direct path from perceived course climate to continuing biology learning intention was estimated simultaneously with these pathways.

Because all variables were measured at a single time point, these analyses were used to characterize statistical indirect-association patterns rather than to establish temporal or causal mediation. A non-significant direct path accompanied by a statistically supported indirect association was interpreted as being consistent with an indirect-only mediation pattern within the specified SEM, traditionally described as full mediation. This terminology refers only to the statistical pattern of coefficients in the present cross-sectional model and does not establish that the proposed mediating sequence occurs causally or over time.

### 3.11 Multi-group SEM by institution type

Multi-group SEM was conducted to examine whether the measurement and structural patterns were broadly comparable across the four participating institution-type groups: comprehensive university, teacher education university, applied undergraduate university, and agricultural/life-science university. Invariance testing proceeded sequentially. Configural invariance was first examined to evaluate whether the same general factor structure was supported across groups. Metric invariance was then evaluated by constraining factor loadings across groups, followed by scalar invariance to assess whether latent means could be compared cautiously. Finally, a structural-path-constrained model was estimated to examine whether the principal structural associations were broadly comparable across groups.

Changes in CFI and RMSEA were used as practical criteria for evaluating increasingly constrained models. A decrease in CFI of approximately 0.010 or less was treated as evidence that the additional constraints did not substantially worsen model fit, with changes in RMSEA also considered (Cheung and Rensvold, 2002; Putnick and Bornstein, 2016).

The group sizes were unequal, with *n* = 247, 118, 114, and 145 across the four groups. This imbalance was considered when interpreting the multi-group results because estimates from the smaller groups may have larger standard errors, wider confidence intervals, and lower statistical power than estimates from the largest group. Accordingly, overall invariance was evaluated primarily from changes in model-fit indices across constrained models, whereas group-specific structural coefficients were treated as exploratory. Differences in statistical significance across groups were not interpreted by themselves as evidence that the corresponding structural parameters differed significantly between groups.

### 3.12 Statistical reporting

Data screening, descriptive statistics, and Pearson correlation analyses were conducted using IBM SPSS Statistics 29.0 (IBM Corp., Armonk, NY, United States). Reliability estimates, including Cronbach’s alpha and McDonald’s omega, were cross-checked in R 4.3.2 using the psych package. Confirmatory factor analysis, structural equation modeling, bootstrapped indirect-association analysis, and multi-group SEM were conducted using Mplus 8.8.

Since the questionnaire items were based on ordered five-point Likert-type response categories, CFA and SEM estimates were made based on an ordinal method that was compatible with WLSMV. In case of indirect relationships, 95% confidence intervals were calculated based on 5,000 bootstrap resamples. The model fit was assessed through the value of chi-square, degrees of freedom (df), comparative fit index (CFI), Tucker-Lewis index (TLI), root mean square error of approximation (RMSEA), and standardized root mean residual (SRMR). Changes in CFI and RMSEA were employed as practical guidelines in assessing invariance in multi-group SEM.

Statistical tests were all two-tailed unless stated differently. To enable interpretation across constructs, SEM paths were standardized coefficients in order to report them. The statistical significance level was set as *p* < 0.05. Nevertheless, the interpretation focused on the size of the coefficients, confidence intervals, model fit, and compatibility with the theoretical framework instead of solely on statistical significance.

The Results section is organized around the three research questions. RQ1 addresses descriptive statistics, reliability, and validity evidence, and the CFA measurement-model evaluation. RQ2 addresses bivariate associations, structural path estimates, and bootstrapped indirect associations. RQ3 addresses descriptive group profiles, measurement invariance, and exploratory multi-group structural comparisons.

## 4 Results

### 4.1 RQ1: Measurement model and construct validity

RQ1 examined whether the proposed measurement model provided adequate reliability and construct validity evidence for the focal constructs.

#### 4.1.1 Descriptive statistics of the main constructs

Descriptive statistics and reliability estimates for the main constructs are presented in Table 3. Mean scores for the six focal constructs were generally slightly above the midpoint of the five-point response scale. Perceived need-supportive biology course climate had a mean of 3.503 (SD = 0.515), basic psychological needs had a mean of 3.436 (SD = 0.532), biology learning self-efficacy had a mean of 3.392 (SD = 0.632), and science identity had a mean of 3.385 (SD = 0.616). Biology learning engagement had a mean of 3.463 (SD = 0.521), while continuing biology learning intention had a mean of 3.385 (SD = 0.663).

**Table 3:** Descriptive statistics and reliability estimates for the main constructs.

Scale code	Construct	*k*	*M*	SD	α	ω	CR	AVE
AS	Autonomy support	4	3.568	0.612	0.713	0.788	0.788	0.482
CS	Competence support	4	3.475	0.627	0.717	0.791	0.791	0.485
RS	Relatedness support	4	3.466	0.601	0.681	0.773	0.773	0.459
Course climate	Need-supportive biology course climate	12	3.503	0.515	0.848	0.916	0.916	0.476
AUT	Autonomy need satisfaction	4	3.425	0.637	0.728	0.796	0.796	0.494
COM	Competence need satisfaction	4	3.399	0.641	0.725	0.795	0.795	0.492
REL	Relatedness needs satisfaction	4	3.483	0.646	0.733	0.798	0.798	0.498
Basic needs	Basic psychological needs	12	3.436	0.532	0.851	0.922	0.922	0.495
BSE	Biology learning self-efficacy	5	3.392	0.632	0.776	0.818	0.818	0.473
SI	Science identity	5	3.385	0.616	0.751	0.804	0.804	0.451
BE	Behavioral engagement	4	3.517	0.645	0.718	0.791	0.791	0.486
CEG	Cognitive engagement	4	3.389	0.640	0.699	0.781	0.781	0.472
EE	Emotional engagement	4	3.483	0.622	0.721	0.792	0.792	0.488
Engagement	Biology learning engagement	12	3.463	0.521	0.839	0.918	0.918	0.482
CBLI	Continuing biology Learning intention	4	3.385	0.663	0.735	0.800	0.800	0.500

*N* = 624. All substantive items were rated from 1 = strongly disagree to 5 = strongly agree. Higher scores indicate higher levels of the construct. α = Cronbach’s alpha; ω = McDonald’s omega; CR, composite reliability; AVE, average variance extracted.

Facet-level means were also close to or slightly above the scale midpoint. Within perceived need-supportive biology course climate, autonomy support had the highest mean (*M* = 3.568, SD = 0.612), followed by competence support (*M* = 3.475, SD = 0.627) and relatedness support (*M* = 3.466, SD = 0.601). Within basic psychological needs, relatedness need satisfaction had the highest mean (*M* = 3.483, SD = 0.646), followed by autonomy need satisfaction (*M* = 3.425, SD = 0.637) and competence need satisfaction (*M* = 3.399, SD = 0.641). Within biology learning engagement, behavioral engagement had the highest mean (*M* = 3.517, SD = 0.645), followed by emotional engagement (*M* = 3.483, SD = 0.622) and cognitive engagement (*M* = 3.389, SD = 0.640).

The scale-level distributions did not show severe univariate departures from normality. For the six focal constructs, skewness ranged from −0.166 to −0.005, and kurtosis ranged from −0.456 to −0.083. At the item level, floor percentages were low, ranging from 0.2 to 2.4%, while ceiling percentages ranged from 7.1 to 14.9%. Full item-level descriptive statistics are reported in Supplementary Table S1. No missing values were observed for the analyzed items or scale scores.

#### 4.1.2 Reliability and convergent validity

Internal consistency estimates were acceptable for the focal constructs. Cronbach’s alpha was 0.848 for perceived need-supportive biology course climate, 0.851 for basic psychological needs, 0.839 for biology learning engagement, and 0.735 for continuing biology learning intention. McDonald’s omega values were 0.916, 0.922, 0.918, and 0.800, respectively. Biology learning self-efficacy showed *α* = 0.776 and *ω* = 0.818, while science identity showed α = 0.751 and ω = 0.804.

Facet-level reliability estimates were also within an acceptable range for exploratory educational psychology research. Cronbach’s alpha ranged from 0.681 for relatedness support to 0.733 for relatedness need satisfaction. McDonald’s omega ranged from 0.773 to 0.798 across the first-order facets. Composite reliability values ranged from 0.773 to 0.922. AVE estimates ranged from 0.451 to 0.500. Although several AVE values were slightly below 0.50, the combination of standardized loadings, composite reliability, and omega estimates provided acceptable evidence for proceeding with the structural analysis.

#### 4.1.3 Confirmatory factor analysis

Four measurement models were compared (Table 4). The one-factor model yielded χ^2^ = 5,726.34, df = 1,175, *p* < 0.001, CFI = 0.742, TLI = 0.728, RMSEA = 0.079, 90% CI (0.077, 0.081), and SRMR = 0.084, indicating that the item covariance structure was not adequately represented by a single common factor. This comparison was treated only as a limited diagnostic and was not interpreted as ruling out common method variance. The six-factor construct-level model yielded χ^2^ = 2,575.20, df = 1,160, *p* < 0.001, CFI = 0.920, TLI = 0.914, RMSEA = 0.044, 90% CI (0.042, 0.046), and SRMR = 0.053. The higher-order measurement model yielded χ^2^ = 2,248.12, df = 1,147, *p* < 0.001, CFI = 0.938, TLI = 0.932, RMSEA = 0.039, 90% CI (0.037, 0.041), and SRMR = 0.047.

**Table 4:** Confirmatory factor analysis model fit comparison.

Model	Estimator	χ^2^	df	*p*	CFI	TLI	RMSEA	90% CI for RMSEA	SRMR	Interpretation
M1: one-factor model	WLSMV-compatible ordinal CFA	5,726.34	1,175	< 0.001	0.742	0.728	0.079	[0.077, 0.081]	0.084	Poor fit; discriminant structure not supported
M2: six-factor construct-level model	WLSMV-compatible ordinal CFA	2,575.20	1,160	< 0.001	0.920	0.914	0.044	[0.042, 0.046]	0.053	Acceptable fit
M3: correlated first-order measurement model	WLSMV-compatible ordinal CFA	2,193.75	1,139	< 0.001	0.941	0.934	0.039	[0.037, 0.041]	0.045	Preferred measurement model
M4: higher-order measurement model	WLSMV-compatible ordinal CFA	2,248.12	1,147	< 0.001	0.938	0.932	0.039	[0.037, 0.041]	0.047	Parsimonious alternative

CFA, confirmatory factor analysis; CFI, comparative fit index; TLI, Tucker–Lewis index; RMSEA, root mean square error of approximation; SRMR, standardized root mean square residual. Because the CFA models were estimated using a WLSMV-compatible ordinal approach, AIC and BIC were not used for model comparison.

Among the models compared, the correlated first-order measurement model provided the most favorable overall pattern of fit indices, χ^2^ = 2,193.75, df = 1,139, *p* < 0.001, CFI = 0.941, TLI = 0.934, RMSEA = 0.039, 90% CI (0.037, 0.041), and SRMR = 0.045. Although its CFI and TLI values remained below the more stringent 0.95 reference value, its RMSEA and SRMR values were comparatively favorable, and it provided the best relative fit among the theoretically specified models examined. This model was therefore retained as the preferred measurement model for subsequent analyses.

### 4.2 RQ2: Structural and indirect associations

RQ2 examined the direct and indirect statistical associations among perceived need-supportive biology course climate, basic psychological needs, biology learning self-efficacy, science identity, biology learning engagement, and continuing biology learning intention.

#### 4.2.1 Correlations among the main constructs

Table 5 presents the Pearson correlations among the six focal constructs. All correlations were positive and statistically significant at *p* < 0.001. The strongest bivariate association was observed between perceived need-supportive biology course climate and basic psychological needs (*r* = 0.553). Basic psychological needs were also strongly correlated with biology learning engagement (*r* = 0.535) and continuing biology learning intention (*r* = 0.470).

**Table 5:** Pearson correlation matrix among the main constructs.

Construct	1	2	3	4	5	6
1. Need-supportive biology course climate	1.000					
2. Basic psychological needs	0.553	1.000				
3. Biology learning self-efficacy	0.371	0.420	1.000			
4. Science identity	0.369	0.447	0.376	1.000		
5. Biology learning engagement	0.449	0.535	0.436	0.482	1.000	
6. Continuing biology learning intention	0.370	0.470	0.391	0.450	0.507	1.000

*N* = 624. All correlations were statistically significant at *p* < 0.001. Values are Pearson correlation coefficients.

Biology learning engagement was positively correlated with continuing biology learning intention (*r* = 0.507), science identity (*r* = 0.482), biology learning self-efficacy (*r* = 0.436), basic psychological needs (*r* = 0.535), and perceived course climate (*r* = 0.449). Science identity was positively correlated with continuing biology learning intention (*r* = 0.450), and biology learning self-efficacy was positively correlated with continuing biology learning intention (*r* = 0.391). Overall, the correlation coefficients ranged from *r* = 0.369 to *r* = 0.553, indicating moderate positive associations among the study constructs.

#### 4.2.2 Structural equation model

**Figure 2:**
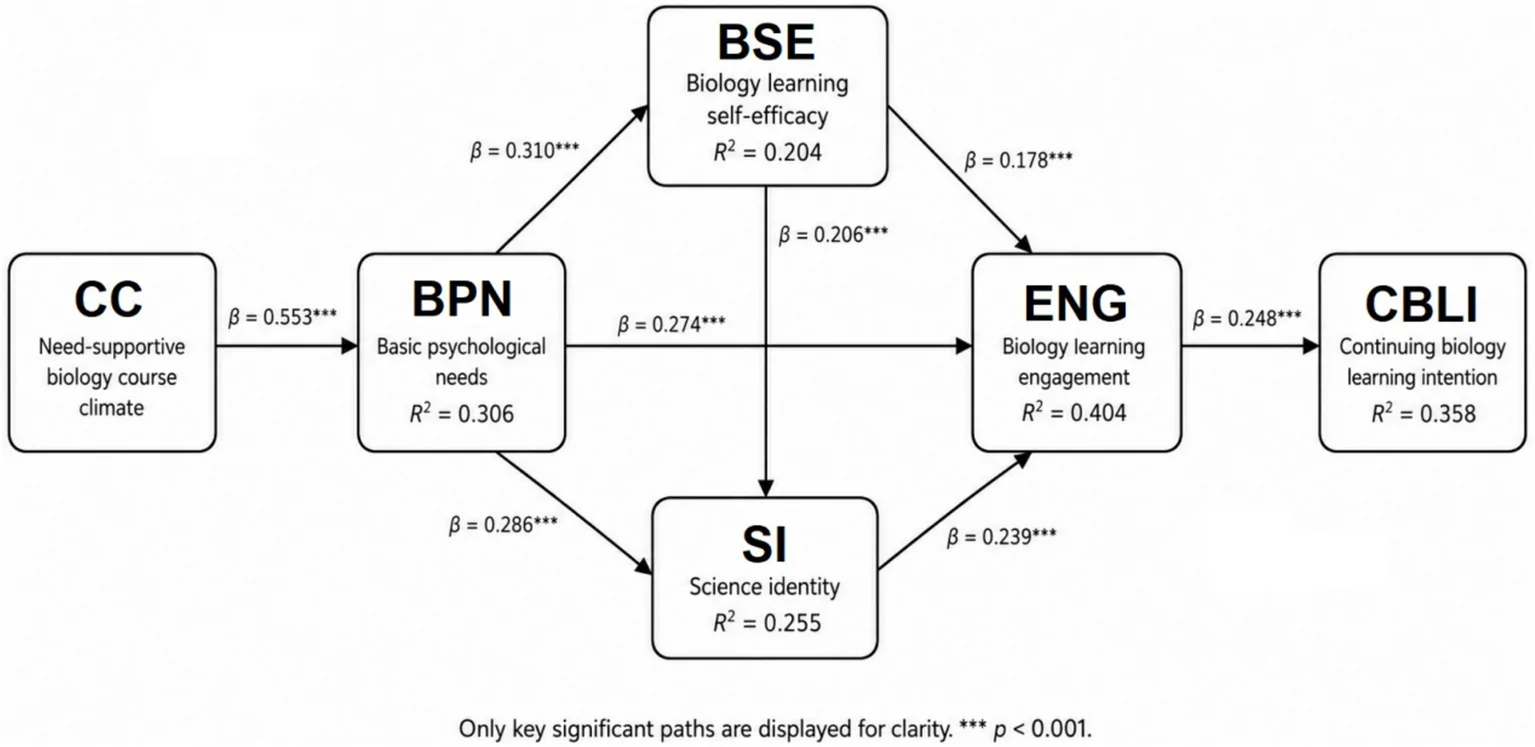
Final standardized structural equation model. CC, perceived need-supportive biology course climate; BPN, basic psychological needs; BSE, biology learning self-efficacy; SI, science identity; ENG, biology learning engagement; CBLI, continuing biology learning intention. Standardized path coefficients are shown on arrows. Solid arrows indicate statistically clear paths; the dashed arrow indicates the non-significant direct path from perceived course climate to continuing biology learning intention. *R*^2^ values are shown inside or below endogenous constructs. ***p < 0.001, **p < 0.01, ns, not statistically significant. Because the study used cross-sectional self-report data, the paths represent statistical associations rather than causal effects.

The proposed structural equation model yielded χ^2^ = 2,354.62, df = 1,166, *p* < 0.001, CFI = 0.935, TLI = 0.929, RMSEA = 0.040, 90% CI (0.038, 0.042), and SRMR = 0.048. The CFI and TLI values were below the more stringent 0.95 reference value, whereas the RMSEA and SRMR values were comparatively favorable. Accordingly, the global fit indices were interpreted as providing mixed evidence regarding overall model fit rather than as indicating uniformly strong fit. Standardized path coefficients are reported in Table 6 and displayed in Figure 2.

**Table 6:** Standardized structural equation model path results.

Endogenous construct	Predictor	*β*	SE	*z*	*p*	95% CI	*R* ^2^	Interpretation
Basic psychological needs	Need-supportive biology course climate	0.553	0.033	16.55	< 0.001	[0.487, 0.618]	0.306	Positive
Biology learning self-efficacy	Need-supportive biology course climate	0.199	0.043	4.64	<0.001	[0.115, 0.284]	0.204	Positive
Biology learning self-efficacy	Basic psychological needs	0.310	0.043	7.22	<0.001	[0.226, 0.394]	0.204	Positive
Science identity	Need-supportive biology course climate	0.135	0.042	3.19	0.002	[0.052, 0.218]	0.255	Positive
Science identity	Basic psychological needs	0.286	0.043	6.60	<0.001	[0.201, 0.371]	0.255	Positive
Science identity	Biology learning self-efficacy	0.206	0.039	5.31	<0.001	[0.130, 0.283]	0.255	Positive
Biology learning engagement	Need-supportive biology course climate	0.143	0.038	3.75	<0.001	[0.068, 0.218]	0.404	Positive
Biology learning engagement	Basic psychological needs	0.274	0.040	6.83	<0.001	[0.195, 0.353]	0.404	Positive
Biology learning engagement	Biology learning self-efficacy	0.178	0.036	5.01	<0.001	[0.108, 0.248]	0.404	Positive
Biology learning engagement	Science identity	0.239	0.036	6.65	<0.001	[0.169, 0.310]	0.404	Positive
Continuing biology learning intention	Need-supportive biology course climate	0.047	0.040	1.17	0.243	[−0.032, 0.126]	0.358	Not statistically clear
Continuing biology learning intention	Basic psychological needs	0.176	0.043	4.08	<0.001	[0.092, 0.261]	0.358	Positive
Continuing biology learning intention	Biology learning self-efficacy	0.120	0.038	3.19	0.001	[0.046, 0.194]	0.358	Positive
Continuing biology learning intention	Science identity	0.189	0.039	4.89	<0.001	[0.113, 0.265]	0.358	Positive
Continuing biology learning intention	Biology learning engagement	0.248	0.042	5.94	<0.001	[0.166, 0.330]	0.358	Positive

*N* = 624. Standardized coefficients are reported. CI, confidence interval. *R*^2^ values refer to the explained variance of each endogenous construct. Global SEM fit: χ^2^ = 2,354.62, df = 1,166, *p* < 0.001, CFI = 0.935, TLI = 0.929, RMSEA = 0.040, 90% CI [0.038, 0.042], SRMR = 0.048.

Perceived need-supportive biology course climate was positively associated with basic psychological needs, *β* = 0.553, SE = 0.033, *z* = 16.55, *p* < 0.001, 95% CI (0.487, 0.618). The model explained 30.6% of the variance in basic psychological needs.

Biology learning self-efficacy was positively associated with both perceived course climate, *β* = 0.199, SE = 0.043, *z* = 4.64, *p* < 0.001, 95% CI (0.115, 0.284), and basic psychological needs, *β* = 0.310, SE = 0.043, *z* = 7.22, *p* < 0.001, 95% CI (0.226, 0.394). Together, these predictors explained 20.4% of the variance in biology learning self-efficacy.

Science identity was positively associated with perceived course climate, *β* = 0.135, SE = 0.042, *z* = 3.19, *p* = 0.002, 95% CI (0.052, 0.218), basic psychological needs, *β* = 0.286, SE = 0.043, *z* = 6.60, *p* < 0.001, 95% CI (0.201, 0.371), and biology learning self-efficacy, *β* = 0.206, SE = 0.039, *z* = 5.31, *p* < 0.001, 95% CI (0.130, 0.283). The model explained 25.5% of the variance in science identity.

Biology learning engagement was positively associated with perceived course climate, *β* = 0.143, SE = 0.038, *z* = 3.75, *p* < 0.001, 95% CI (0.068, 0.218), basic psychological needs, *β* = 0.274, SE = 0.040, *z* = 6.83, *p* < 0.001, 95% CI (0.195, 0.353), biology learning self-efficacy, *β* = 0.178, SE = 0.036, *z* = 5.01, *p* < 0.001, 95% CI (0.108, 0.248), and science identity, *β* = 0.239, SE = 0.036, *z* = 6.65, *p* < 0.001, 95% CI (0.169, 0.310). The model explained 40.4% of the variance in biology learning engagement.

Continuing biology learning intention was positively associated with basic psychological needs, *β* = 0.176, SE = 0.043, *z* = 4.08, *p* < 0.001, 95% CI (0.092, 0.261), biology learning self-efficacy, *β* = 0.120, SE = 0.038, *z* = 3.19, *p* = 0.001, 95% CI (0.046, 0.194), science identity, *β* = 0.189, SE = 0.039, *z* = 4.89, *p* < 0.001, 95% CI (0.113, 0.265), and biology learning engagement, *β* = 0.248, SE = 0.042, *z* = 5.94, *p* < 0.001, 95% CI (0.166, 0.330). The direct association between perceived course climate and continuing biology learning intention was not statistically clear after the mediating variables were included, *β* = 0.047, SE = 0.040, *z* = 1.17, *p* = 0.243, 95% CI (−0.032, 0.126). The model explained 35.8% of the variance in continuing biology learning intention.

#### 4.2.3 Bootstrapped indirect associations

Table 7 presents the bootstrapped indirect associations based on 5,000 resamples. All tested indirect associations had 95% bootstrapped confidence intervals that did not include zero.

**Table 7:** Bootstrapped indirect associations.

Indirect association	Point estimate	Bootstrap SE	95% bootstrap CI	Bootstrap samples	Decision
Course climate → basic needs → learning engagement	0.151	0.023	[0.108, 0.199]	5,000	Supported
Course climate → basic needs → self-efficacy → learning engagement	0.031	0.007	[0.017, 0.046]	5,000	Supported
Course climate → basic needs → science identity → learning engagement	0.038	0.008	[0.023, 0.055]	5,000	Supported
Course climate → basic needs → learning engagement → continuing intention	0.038	0.009	[0.022, 0.057]	5,000	Supported
Basic needs → learning engagement → continuing intention	0.068	0.016	[0.040, 0.101]	5,000	Supported
Science identity → learning engagement → continuing intention	0.059	0.013	[0.035, 0.087]	5,000	Supported
Self-efficacy → learning engagement → continuing intention	0.044	0.012	[0.024, 0.070]	5,000	Supported

Indirect associations were considered statistically supported when the 95% bootstrapped confidence interval did not include zero. Estimates are standardized indirect associations. Because the study was cross-sectional, these estimates characterize statistical indirect-association patterns and should not be interpreted as evidence of temporal or causal mediation.

Perceived need-supportive biology course climate was indirectly associated with biology learning engagement through basic psychological needs, point estimate = 0.151, SE = 0.023, 95% CI (0.108, 0.199). It was also indirectly associated with learning engagement through basic psychological needs and biology learning self-efficacy, point estimate = 0.031, SE = 0.007, 95% CI (0.017, 0.046), and through basic psychological needs and science identity, point estimate = 0.038, SE = 0.008, 95% CI (0.023, 0.055).

Perceived course climate was indirectly associated with continuing biology learning intention through basic psychological needs and learning engagement, point estimate = 0.038, SE = 0.009, 95% CI (0.022, 0.057). Basic psychological needs were indirectly associated with continuing intention through learning engagement, point estimate = 0.068, SE = 0.016, 95% CI (0.040, 0.101). Biology learning self-efficacy was indirectly associated with continuing intention through learning engagement, point estimate = 0.044, SE = 0.012, 95% CI (0.024, 0.070). Science identity was also indirectly associated with continuing intention through learning engagement, point estimate = 0.059, SE = 0.013, 95% CI (0.035, 0.087).

Perceived need-supportive biology course climate was indirectly associated with continuing biology learning intention through basic psychological needs and learning engagement, with a standardized indirect estimate of 0.038 [SE = 0.009, 95% bootstrap CI (0.022, 0.057)]. At the same time, the direct association between perceived course climate and continuing biology learning intention was not statistically clear after the motivational and engagement-related variables were included [*β* = 0.047, *p* = 0.243, 95% CI (−0.032, 0.126)]. Within the specified SEM, the combination of a statistically supported indirect association and a non-significant direct path is consistent with an indirect-only mediation pattern, traditionally referred to as full mediation. However, because all variables were measured cross-sectionally, this designation describes only the statistical pattern of associations and should not be interpreted as evidence of causal or temporal mediation.

### 4.3 RQ3: Comparisons across participating institution-type groups

RQ3 examined whether the measurement properties and structural associations were broadly comparable across the participating institution-type groups. Group mean profiles are presented first as descriptive context, followed by measurement-invariance and multi-group structural analyses.

#### 4.3.1 Group mean profiles by institution type

**Figure 3:**
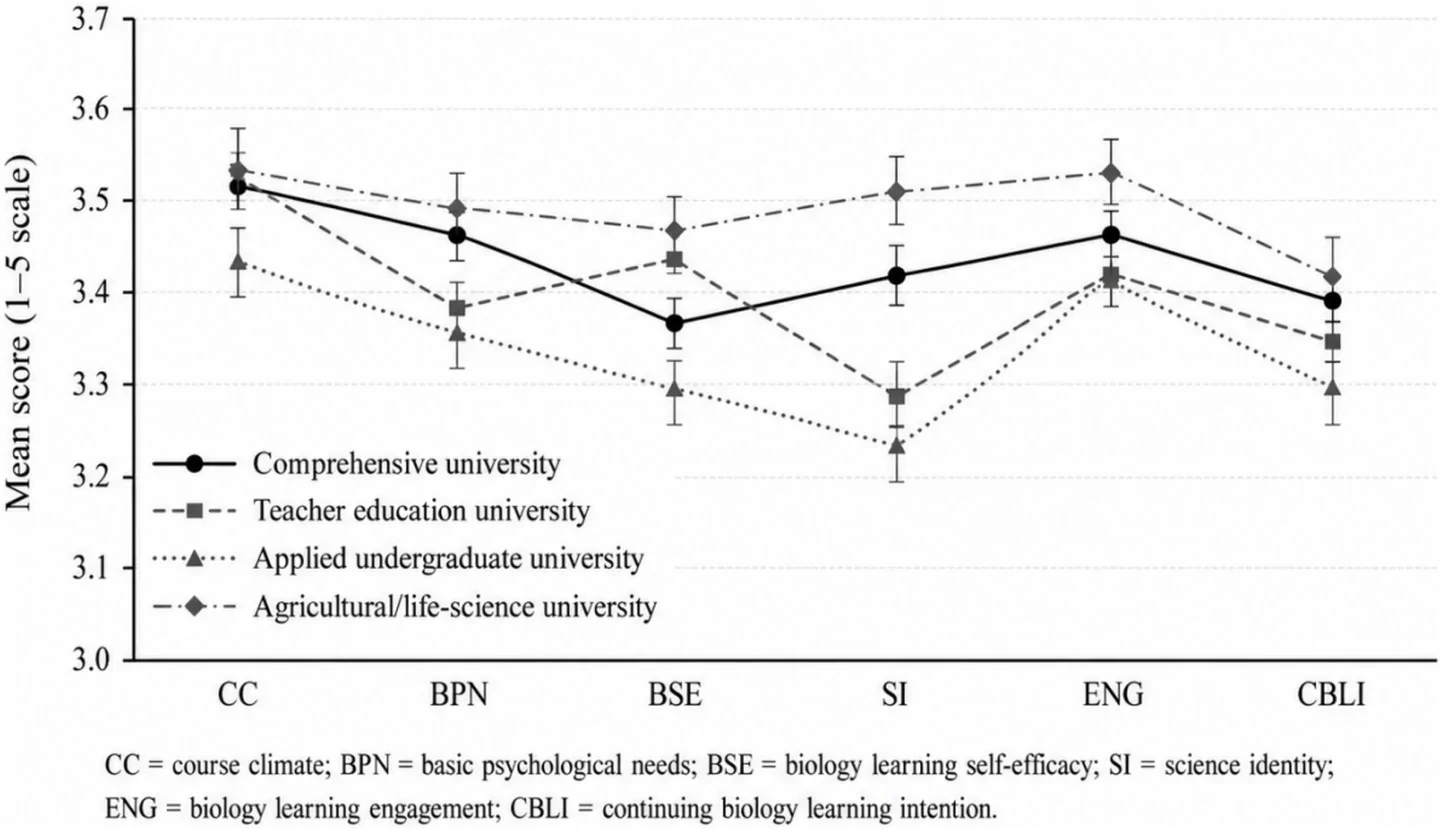
Group mean profiles of the main constructs by institution type. The figure displays mean scores for perceived need-supportive biology course climate, basic psychological needs, biology learning self-efficacy, science identity, biology learning engagement, and continuing biology learning intention across four institution types. Error bars represent standard errors. Institution type was used as a contextual comparison variable; the figure is descriptive and does not imply causal differences among institution types.

Figure 3 summarizes the mean profiles of the six main constructs across institution types. The descriptive means were broadly similar across groups, with all group means falling between 3.263 and 3.530. Perceived need-supportive biology course climate ranged from 3.433 in applied undergraduate universities to 3.527 in agricultural/life-science universities. Basic psychological needs ranged from 3.363 in applied undergraduate universities to 3.488 in agricultural/life-science universities.

Agricultural/life-science universities showed the highest descriptive means for biology learning self-efficacy (*M* = 3.465, SD = 0.654), science identity (*M* = 3.512, SD = 0.620), biology learning engagement (*M* = 3.530, SD = 0.531), and continuing biology learning intention (*M* = 3.428, SD = 0.626). Applied undergraduate universities showed the lowest descriptive means for perceived course climate (*M* = 3.433, SD = 0.553), basic psychological needs (*M* = 3.363, SD = 0.542), biology learning self-efficacy (*M* = 3.305, SD = 0.651), science identity (*M* = 3.263, SD = 0.652), and continuing biology learning intention (*M* = 3.329, SD = 0.730). These descriptive differences were interpreted cautiously because institution type was treated as a contextual grouping variable rather than a causal predictor. The group means and standard errors used to generate Figure 3 are reported in Supplementary Table S3.

#### 4.3.2 Multi-group SEM by institution type

Multi-group SEM was conducted to examine whether the measurement and structural patterns were broadly comparable across the participating groups. The configural model yielded χ^2^ = 3,974.52, df = 1,128, CFI = 0.947, TLI = 0.941, RMSEA = 0.042, and SRMR = 0.052. The metric model yielded χ^2^ = 4,049.10, df = 1,161, CFI = 0.945, TLI = 0.940, RMSEA = 0.041, and SRMR = 0.054. Relative to the configural model, the changes were small (ΔCFI = −0.002; ΔRMSEA = −0.001).

The scalar model yielded χ^2^ = 4,138.34, df = 1,194, CFI = 0.941, TLI = 0.936, RMSEA = 0.040, and SRMR = 0.057, with similarly small changes from the metric model (ΔCFI = −0.004; ΔRMSEA = −0.001). The structural-path-constrained model yielded χ^2^ = 4,196.82, df = 1,215, CFI = 0.938, TLI = 0.934, RMSEA = 0.040, and SRMR = 0.059. The change from the scalar model was again small (ΔCFI = −0.003; ΔRMSEA = 0.000). Taken together, the small changes in CFI and RMSEA across increasingly constrained models supported cautious comparison of the measurement and structural patterns across the participating groups (Table 8).

**Table 8:** Multi-group SEM by institution type.

Model	Groups	χ^2^	df	CFI	TLI	RMSEA	SRMR	ΔCFI	ΔRMSEA	Interpretation
Configural invariance	Institution type	3,974.52	1,128	0.947	0.941	0.042	0.052	–	–	Same factor pattern acceptable
Metric invariance	Institution type	4,049.10	1,161	0.945	0.940	0.041	0.054	−0.002	−0.001	Factor loadings comparable
Scalar invariance	Institution type	4,138.34	1,194	0.941	0.936	0.040	0.057	−0.004	−0.001	Latent means can be compared cautiously
Structural paths constrained	Institution type	4,196.82	1,215	0.938	0.934	0.040	0.059	−0.003	0.000	Structural paths broadly comparable

Institution type included comprehensive university, teacher education university, applied undergraduate university, and agricultural/life-science university. ΔCFI and ΔRMSEA were calculated relative to the previous, less constrained model. For the configural model, ΔCFI and ΔRMSEA are not applicable. Group-specific path estimates are reported in Supplementary Table S4.

The path from perceived course climate to basic psychological needs was positive and statistically clear in all institution types: comprehensive universities, *β* = 0.479, *p* < 0.001, 95% CI (0.369, 0.590); teacher education universities, *β* = 0.556, *p* < 0.001, 95% CI (0.403, 0.709); applied undergraduate universities, *β* = 0.597, *p* < 0.001, 95% CI (0.447, 0.747); and agricultural/life-science universities, *β* = 0.630, *p* < 0.001, 95% CI (0.501, 0.758).

The path from basic psychological needs to learning engagement was also positive across all institution types, although the magnitude varied: comprehensive universities, *β* = 0.148, *p* = 0.015; teacher education universities, *β* = 0.523, *p* < 0.001; applied undergraduate universities, *β* = 0.315, *p* = 0.002; and agricultural/life-science universities, *β* = 0.284, *p* = 0.002.

Other group-specific paths showed more variation. Science identity was positively associated with learning engagement in comprehensive universities, *β* = 0.224, *p* < 0.001; applied undergraduate universities, *β* = 0.343, *p* < 0.001; and agricultural/life-science universities, *β* = 0.360, *p* < 0.001. This path was not statistically clear in teacher education universities, *β* = 0.004, *p* = 0.959. Biology learning self-efficacy was positively associated with learning engagement in comprehensive universities, *β* = 0.226, *p* < 0.001, and teacher education universities, *β* = 0.196, *p* = 0.020, but not statistically clear in applied undergraduate universities, *β* = 0.108, *p* = 0.168, or agricultural/life-science universities, *β* = 0.127, *p* = 0.100.

Learning engagement was positively associated with continuing biology learning intention in comprehensive universities, *β* = 0.306, *p* < 0.001; teacher education universities, *β* = 0.202, *p* = 0.046; and applied undergraduate universities, *β* = 0.336, *p* < 0.001. This path was not statistically clear in agricultural/life-science universities, *β* = 0.150, *p* = 0.102. These group-specific estimates were treated as exploratory. Because the group sizes were unequal, the smaller groups may have had less statistical power and less precise parameter estimates, as reflected in potentially larger standard errors and wider confidence intervals. Therefore, differences in whether individual paths reached statistical significance across groups were not interpreted as formal evidence of between-group differences in the corresponding structural coefficients. The small changes in global fit indices for the structural-path-constrained model instead suggested broad comparability of the overall structural pattern, while the individual group-specific estimates were retained primarily as descriptive information.

### 4.4 Summary of results

The measurement analyses showed that the correlated first-order model provided the most favorable fit among the measurement models examined. Its RMSEA and SRMR values were comparatively favorable, although the CFI and TLI remained below the more stringent 0.95 reference value. The focal constructs also showed generally adequate internal consistency. Correlation analyses indicated positive associations among perceived need-supportive biology course climate, basic psychological needs, biology learning self-efficacy, science identity, biology learning engagement, and continuing biology learning intention.

In the structural model, perceived need-supportive biology course climate was positively associated with basic psychological needs. Basic psychological needs, biology learning self-efficacy, and science identity were positively associated with biology learning engagement, which in turn was positively associated with continuing biology learning intention. The direct path from perceived course climate to continuing biology learning intention was not statistically significant after the motivational and engagement-related variables were included, whereas several bootstrapped indirect associations were supported. In the multi-group analyses, changes in CFI and RMSEA across increasingly constrained models were small, supporting cautious comparison across the participating groups, although group-specific structural estimates remained exploratory.

## 5 Discussion

### 5.1 Principal findings

This study examined an integrated self-determination theory-based model of biology learning among Chinese university students. The model linked students’ perceived need-supportive biology course climate with basic psychological needs, biology learning self-efficacy, science identity, biology learning engagement, and continuing biology learning intention. The correlated first-order measurement model provided the most favorable fit among the measurement models examined. Its RMSEA and SRMR values were comparatively favorable, whereas the CFI and TLI remained below the more stringent 0.95 reference value. The structural model showed a similar pattern of fit indices; therefore, the results are interpreted with appropriate caution rather than as evidence of uniformly strong model fit.

The structural estimates showed that perceived need-supportive biology course climate was positively associated with basic psychological needs. Basic psychological needs were positively associated with biology learning self-efficacy, science identity, and learning engagement. Biology learning self-efficacy and science identity were also positively associated with engagement, and engagement was positively associated with continuing biology learning intention.

A notable pattern was that the direct association between perceived course climate and continuing biology learning intention was not statistically clear after motivational and engagement-related variables were included, whereas several bootstrapped indirect associations were supported. This pattern is consistent with an indirect-association structure in which perceived course climate is related to continuing learning intention through more proximal motivational and engagement-related variables. Because the study was cross-sectional, these findings represent statistical associations and should not be interpreted as evidence of causal mediation or temporal ordering.

The multi-group analyses showed only small changes in CFI and RMSEA across increasingly constrained models, supporting cautious comparison of the measurement and structural patterns across the participating groups. However, group-specific path estimates are treated as exploratory, particularly given the unequal group sizes and the limited institutional representation within some categories.

### 5.2 Perceived need-supportive course climate and basic psychological needs

The most powerful structural relationship in the model was the connection between the perceived need-supportive climate of biology courses and basic psychological needs. This result is in line with the self-determination theory, which posits that autonomy, competence, and relatedness are fundamental psychological requirements that are developed through social and schooling conditions (Deci and Ryan, 2000; Ryan and Deci, 2000, 2017; Vansteenkiste et al., 2020). In the current research, students who experienced a higher level of autonomy, competence, and relatedness in their biology courses also indicated greater levels of satisfaction of their basic psychological needs.

It is consistent with previous studies in education that claim autonomy support, structure, and interpersonal involvement are important aspects of the students’ motivational functioning and engagement (Skinner and Belmont, 1993; Reeve et al., 2004; Jang et al., 2010; Niemiec and Ryan, 2009). The implication of this on a biology-learning environment may be that students can take advantage when they feel that the course offers them meaningful options, clear direction, constructive criticism, and friendly interactions. Such characteristics could assist students in perceiving biology learning as more self-endorsed, manageable, and socially related.

The finding can also be theoretically valuable since it places the concept of course climate in the category of a perception of learning environment as opposed to an objective measure of teaching quality. The data cannot indicate that a specific course design led to the fulfillment of needs. On the contrary, it indicates that student perceptions of support received in biology classes correlated highly with their motivational experiences.

### 5.3 The roles of biology learning, self-efficacy, and science identity

The model also demonstrated that fundamental psychological requirements were favorably correlated with biology learning self-efficacy and science identity. This trend can be explained by the concept of a student feeling autonomous, competent, and related within a learning environment, having a higher likelihood of developing adaptive learning beliefs and science-related self-understanding. The self-efficacy theory focuses on the belief that students have in their ability to complete particular learning tasks (Bandura, 1977, 1997; Pajares, 1996). Biology learning self-efficacy was used in this article to indicate the level of confidence of the students regarding the understanding of a given biological concept, application of biological knowledge, analysis of difficult questions, and enhancement through learning strategies.

Science identity was a specific aspect of the process. Although self-efficacy is about perceived capability, science identity is about whether students regard science as part of who they are and how they are viewed by others (Carlone and Johnson, 2007; Hazari et al., 2010). The fact that science identity is positively associated with learning engagement implies that children might become more involved in the process of acquiring biology knowledge when the connection is made between competence beliefs and self-concept. The given interpretation is in accord with studies in the field of science education, indicating that self-efficacy and identity are correlated with science commitment, persistence, and involvement in the learning process (Chemers et al., 2011; Robnett et al., 2015; Trujillo and Tanner, 2014).

The results of the SEM showed that both the learning self-efficacy of biology and science identity had a positive correlation with the level of engagement in biology learning. It implies that there are two alternative psychological pathways of engaging in it. Engagement might be facilitated by self-efficacy since learners have confidence that they can perform their tasks related to biology learning. The sense of engagement might be promoted by science identity since students perceive the process of biology or science learning as personally significant. These two ways should not be equated to the same mechanism.

### 5.4 Biology learning engagement as a proximal correlate of continuing intention

A positive correlation between the engagement in learning biology and continuing to learn biology existed. The finding is in line with multidimensional perspectives on engagement, which involve behavioral engagement, investment in cognition, and emotional engagement (Fredricks et al., 2004; Appleton et al., 2006; Reeve, 2012; Sinatra et al., 2015). As part of the present study, those students who had a greater level of behavioral, cognitive, and emotional engagement in the process of biology learning were more inclined to state the intention of further studying the knowledge related to biology.

The result is important since continuing biology learning intention was viewed as a distal self-reported outcome instead of as future behavior. There has been previous research by STEM that has indicated that educational experiences, self-efficacy, identity, and values of students are associated with persistence-related outcomes and science-related choices (Tai et al., 2006; Maltese and Tai, 2011; Perez et al., 2014; Stets et al., 2017). This study contributes to this body of literature by demonstrating that engagement, in a biology-course setting, was a proximal correlate of continuing intention even when the other psychological needs, self-efficacy, and science identity were included in the same model.

The theoretical significance of the unclear direct relationship between perceived course climate and continuing intention makes sense. The implication is that the perception of the course climate as positive does not necessarily lead to the formation of continuing learning intentions in students. Instead, perceived course climate can be associated with continuing intention via more proximal motivational and engagement-related conditions. This trend is in line with SDT, which stresses the role of need satisfaction as a mechanism by which social-contextual support is made relevant in terms of motivation (Deci and Ryan, 2000; Vansteenkiste et al., 2020).

### 5.5 Indirect associations and the integrated SDT-based pathway

The bootstrapped analyses provided statistical support for several of the theoretically specified indirect associations. Perceived need-supportive biology course climate was indirectly associated with biology learning engagement through basic psychological needs, through basic psychological needs and biology learning self-efficacy, and through basic psychological needs and science identity. Perceived course climate was also indirectly associated with continuing biology learning intention through basic psychological needs and learning engagement. Together, these results are consistent with the proposed framework in which perceived course climate is related to learning outcomes through more proximal motivational and engagement-related variables.

Of particular relevance, the direct association between perceived course climate and continuing biology learning intention was not statistically clear after the motivational and engagement-related variables were included (*β* = 0.047, *p* = 0.243), whereas the indirect association through basic psychological needs and learning engagement was statistically supported. Within the specified SEM, this combination is consistent with an indirect-only mediation pattern, which is traditionally described as full mediation. We use this terminology only to describe the statistical configuration of the direct and indirect coefficients; it should not be interpreted as evidence that course climate causally influences continuing intention through the proposed sequence.

This distinction is especially important because all variables were measured concurrently. Cross-sectional indirect associations do not establish temporal precedence among perceived course climate, psychological need satisfaction, self-efficacy, science identity, engagement, and continuing intention, and they may not reproduce the magnitude or direction of mediation processes that unfold over time. Longitudinal designs with temporally separated measurements would be required to evaluate temporal mediation, while intervention-based designs would be needed to provide stronger evidence concerning causal mechanisms.

### 5.6 Institution-type comparisons

The multi-group SEM analyses showed relatively small changes in CFI and RMSEA across the configural, metric, scalar, and structural-path-constrained models. This pattern provided practical support for cautious comparison of the measurement and structural patterns across the participating groups (Cheung and Rensvold, 2002; Putnick and Bornstein, 2016). At the same time, the absolute CFI and TLI values were not uniformly above the more stringent 0.95 reference value, so these findings are interpreted as supporting broad comparability rather than perfect equivalence across groups.

The association between perceived course climate and basic psychological needs was positive and statistically clear in all four groups. Other group-specific coefficients varied in magnitude and statistical clarity. For example, the science identity–engagement association was not statistically clear in the teacher education group, and the engagement–continuing intention association was not statistically clear in the agricultural/life-science group, although the corresponding estimates in some other groups were statistically significant. These patterns should not be interpreted as demonstrating formal between-group differences simply because statistical significance differed across groups.

The unequal group sizes are particularly relevant to these comparisons. The comprehensive-university group was larger (*n* = 247) than the teacher education (*n* = 118), applied undergraduate (*n* = 114), and agricultural/life-science (*n* = 145) groups. Estimates from the smaller groups may therefore be associated with lower statistical power and greater sampling uncertainty. For this reason, the group-specific structural coefficients are considered exploratory and descriptive, whereas greater emphasis is placed on the small changes in global fit indices across constrained models. Larger and more balanced samples are needed before conclusions can be drawn about whether particular structural associations systematically differ across institutional contexts.

### 5.7 Theoretical implications

The article makes three contributions to educational psychology. To begin with, it applies the self-determination theory to the study of university biology learning in a Chinese higher education setting. The results indicate that SDT-based differentiation between autonomy, competence, and relatedness support can be helpful in operationalizing the concept of students’ perceived biology course climate.

The second is that the research incorporates SDT along with science education constructs. All five components are not studied as separate entities, but put into a single framework. It is helpful since the learning of biology is not just a motivational process but also a disciplinary identity process. There are higher chances of students engaging more profoundly when they have the feeling of being able to learn biology and also connect it to themselves as science learners.

Thirdly, the results indicate that continued biology learning intention is a distal learning intention that cannot be considered as a direct result of course climate alone. Perceived course climate in this model was related to the continuing intention through fundamental psychological needs, self-efficacy, science identity, and engagement. It can support the view of the stratification of biology learning motivation, where course climate, psychological needs, learning beliefs, identity, engagement, and intention are in different positions in the motivational system.

### 5.8 Practical implications

The results have a number of implications for biology course design and teaching practice. Firstly, autonomy support in students might be helpful for biology courses. It is not necessary to eliminate structure. Rather, teachers may offer valuable reasons behind learning activities, permit students to ask questions, give limited but meaningful options, and relate the notions of biology with those in the academic or personal spheres of students.

Secondly, competence support seems to be pertinent. Learning biology also tends to entail intricate terminology, abstract systems, reasoning based on evidence, and novel problem-solving activities. Competence can be experienced by students, and self-efficacy in biology learning can be enhanced through clear task structure, incremental levels of difficulty, worked examples, constructive feedback, and a chance to view the progress.

To begin with, support for relatedness ought not be considered marginal. Teacher-student interaction that is respectful, peer interaction that is inclusive, and collaborative inquiry can enhance the feeling of community among students in the learning of biology. It is especially important for those students who are not majoring in biology or have limited previous experience in the subject.

The findings also indicate that the biology classes ought not to be focused only on content delivery. Course activities may also contribute to the construction of science identity in students. Some examples are authentic problems in science, talking about how biological knowledge is created, acknowledging the scientific reasoning of students, presenting a variety of science role models, and assisting students in thinking about how the learning of biology is related to their future education, career, or civic life.

Ultimately, engagement must be considered as an important proximal goal. Students will have an increased likelihood of reporting continuous learning intentions if they are involved in the learning of biology behaviorally, cognitively, and emotionally. Hence, course-level support must not focus on enhancing satisfaction alone but also on developing learning environments that promote active participation.

### 5.9 Limitations and future research

Several limitations should be considered. First, the study used a cross-sectional design in which all focal variables were assessed at the same time point. This design prevents empirical establishment of temporal precedence among perceived course climate, basic psychological needs, self-efficacy, science identity, engagement, and continuing intention. More importantly, indirect associations estimated from cross-sectional data should not be assumed to represent mediation processes that unfold over time, because the magnitude and even the pattern of such estimates may differ when temporal ordering is explicitly modeled. Accordingly, the indirect-only pattern identified in the present SEM is interpreted as a statistical description of the observed covariance structure rather than as evidence of a causal mediation mechanism. Future longitudinal research with temporally separated measurements is needed to test whether changes in perceived course climate precede changes in motivational and engagement-related variables and, subsequently, continuing biology learning intention.

Second, all focal constructs were assessed from the same respondents at the same time point using the same self-report questionnaire and response format. This creates the possibility that some of the observed covariance among constructs may reflect shared method characteristics rather than substantive relationships alone. Although the one-factor CFA model showed poor fit, this result only indicates that a single common factor does not adequately reproduce the covariance structure of the items. The one-factor comparison is a limited diagnostic and cannot rule out more complex forms of common method variance or determine the magnitude of method-related bias (Podsakoff et al., 2003, 2012). Similarly, the anonymous survey format and absence of personally identifiable information may reduce evaluation concerns, but do not eliminate common-source or common-occasion effects. Future studies should therefore consider stronger procedural and analytical approaches, including temporal or source separation, multiple-method measurement, marker variables, or explicitly modeled method factors.

The third one, perceived course climate, was not an objective measurement of course design or pedagogical practice. It is demonstrated by the results how student perceptions correlated with motivational and engagement outcomes. They cannot indicate whether particular instructional strategies objectively led to the observed patterns.

The fourth one is that the continuation of the biology learning intention is self-reported. It should not be considered as real future enrollment, persistence, course choice, or occupational behavior. The next generation of studies ought to relate the intention data to longitudinal behavioral results when feasible.

Fifth, the institutions were anonymized, and the sample was not representative of the nation. Although multi-institutional design is better in terms of contextual diversity, results cannot be generalized to all Chinese universities unless replicated.

Sixth, though the reliability evidence was mostly acceptable, a few of the AVE estimates were just under 0.50. It indicates that the convergent validity evidence was sufficient but not conclusive. The measurement model must be validated in independent samples in future studies, and the items refined as appropriate.

Finally, the group sizes used in the multi-group SEM were unequal. The comprehensive-university group was substantially larger than some of the other groups, particularly the applied undergraduate group. Although the global invariance results showed only small changes in fit indices across increasingly constrained models, unequal group sizes can affect the precision and statistical power of group-specific structural estimates. In particular, a non-significant path in a smaller group may reflect greater sampling uncertainty or lower power rather than the absence of an association. The group-specific coefficients should therefore be regarded as exploratory, and differences in statistical significance across groups should not be treated as direct evidence of between-group parameter differences. Future studies with larger and more balanced group samples are needed to evaluate the stability of these patterns.

## 6 Conclusion

In this study, a combined SDT-based model of biology education was evaluated among Chinese university students. The results offer cross-sectional evidence that the perceived need-satisfying biology class environment correlates positively with basic psychological needs, which in turn are related to the self-efficacy of biology learning, science identity, learning activities, and future intent on continuing to learn biology. After including motivational and engagement-related variables, the direct relationship between perceived course climate and continuing intention was not statistically significant, but there were several indirect relationships.

The findings indicate that participation in biology learning and the desire to continue learning are associated with a more extensive motivational framework based on the satisfaction of needs, the perception of capabilities, and the identity of science. The results have implications for biology teachers in that they indicate the importance of course climates that promote autonomy, competence, and relatedness as well as assist students in developing self-efficacy and science identity. The interpretation of these conclusions must take into account the cross-sectional, self-report characteristics of the study. Further longitudinal and intervention research is required to determine the directions of causality and to explore if the changes in course climate result in changes in student motivation, engagement, and continuing biology learning behavior.

## Data Availability

The original contributions presented in the study are included in the article/Supplementary material, further inquiries can be directed to the corresponding author.
