# Supplementary material for: Perceived need-supportive biology course climate, learning engagement, and continuing biology learning intention: a cross-sectional SEM study of Chinese university students
Source: Front Psychol. 2026 Aug 26;17:1913690. doi: 10.3389/fpsyg.2026.1913690 (PMC13553953; doi:10.3389/fpsyg.2026.1913690)
Supplement: Supplementary file 1 [file Supplementary_file_1.docx]

**Supplementary Appendix 1. English-translated survey item wording**

| **Construct** | **Factor** | **Item** | **English item wording** |
| --- | --- | --- | --- |
| Need-Supportive Biology Course Climate | Autonomy support | AS1 | The biology course gives me opportunities to make meaningful choices in learning activities. |
| Need-Supportive Biology Course Climate | Autonomy support | AS2 | The instructor encourages students to express their own ideas about biological topics. |
| Need-Supportive Biology Course Climate | Autonomy support | AS3 | I can decide how to approach some biology learning tasks according to my learning needs. |
| Need-Supportive Biology Course Climate | Autonomy support | AS4 | The course explains why biology learning activities are valuable for students. |
| Need-Supportive Biology Course Climate | Competence support | CS1 | The course provides clear guidance that helps me understand how to improve in biology. |
| Need-Supportive Biology Course Climate | Competence support | CS2 | Feedback in this course helps me feel capable of learning biology. |
| Need-Supportive Biology Course Climate | Competence support | CS3 | The difficulty of biology learning tasks is generally appropriate for my current level. |
| Need-Supportive Biology Course Climate | Competence support | CS4 | The course helps me connect biological concepts with solvable problems. |
| Need-Supportive Biology Course Climate | Relatedness support | RS1 | I feel respected when participating in biology learning activities. |
| Need-Supportive Biology Course Climate | Relatedness support | RS2 | The biology learning environment makes students feel included. |
| Need-Supportive Biology Course Climate | Relatedness support | RS3 | I feel that my classmates and instructor are supportive during biology learning. |
| Need-Supportive Biology Course Climate | Relatedness support | RS4 | The course encourages constructive communication among students. |
| Basic Psychological Needs | Autonomy need satisfaction | AUT1 | I feel that I have a sense of choice when learning biology. |
| Basic Psychological Needs | Autonomy need satisfaction | AUT2 | I can study biology in ways that fit my own learning preferences. |
| Basic Psychological Needs | Autonomy need satisfaction | AUT3 | I feel free to ask questions or share opinions in biology learning. |
| Basic Psychological Needs | Autonomy need satisfaction | AUT4 | Biology learning allows me to connect course content with my own interests. |
| Basic Psychological Needs | Competence need satisfaction | COM1 | I feel capable of understanding important biology concepts. |
| Basic Psychological Needs | Competence need satisfaction | COM2 | I can complete biology learning tasks when I make sufficient effort. |
| Basic Psychological Needs | Competence need satisfaction | COM3 | I can use feedback to improve my biology learning. |
| Basic Psychological Needs | Competence need satisfaction | COM4 | I feel that I am making progress in biology learning. |
| Basic Psychological Needs | Relatedness need satisfaction | REL1 | I feel connected with others during biology learning. |
| Basic Psychological Needs | Relatedness need satisfaction | REL2 | I feel accepted by peers in biology learning activities. |
| Basic Psychological Needs | Relatedness need satisfaction | REL3 | I can cooperate with classmates when learning biology. |
| Basic Psychological Needs | Relatedness need satisfaction | REL4 | I feel that the biology course supports positive learning relationships. |
| Biology Learning Self-Efficacy | Biology learning self-efficacy | BSE1 | I am confident that I can understand core concepts in biology. |
| Biology Learning Self-Efficacy | Biology learning self-efficacy | BSE2 | I can apply biological knowledge to explain unfamiliar phenomena. |
| Biology Learning Self-Efficacy | Biology learning self-efficacy | BSE3 | I can analyze biology-related questions even when they are challenging. |
| Biology Learning Self-Efficacy | Biology learning self-efficacy | BSE4 | I can use evidence to support explanations in biology learning. |
| Biology Learning Self-Efficacy | Biology learning self-efficacy | BSE5 | I can improve my biology performance through effective learning strategies. |
| Science Identity | Science identity | SI1 | I see myself as a person who can learn science well. |
| Science Identity | Science identity | SI2 | Biology learning helps me feel connected to scientific ways of thinking. |
| Science Identity | Science identity | SI3 | People important to me would recognize my effort in science learning. |
| Science Identity | Science identity | SI4 | Learning biology is related to the kind of person I want to become. |
| Science Identity | Science identity | SI5 | I feel that scientific thinking can be part of my academic identity. |
| Biology Learning Engagement | Behavioral engagement | BE1 | I participate actively in biology learning activities. |
| Biology Learning Engagement | Behavioral engagement | BE2 | I complete biology learning tasks with sustained effort. |
| Biology Learning Engagement | Behavioral engagement | BE3 | I prepare for biology learning activities before class or study sessions. |
| Biology Learning Engagement | Behavioral engagement | BE4 | I pay close attention when learning biology. |
| Biology Learning Engagement | Cognitive engagement | CEG1 | I try to connect new biology knowledge with what I have learned before. |
| Biology Learning Engagement | Cognitive engagement | CEG2 | I use strategies to understand difficult biological concepts. |
| Biology Learning Engagement | Cognitive engagement | CEG3 | I think about how different biology concepts are related. |
| Biology Learning Engagement | Cognitive engagement | CEG4 | I reflect on mistakes or misunderstandings in biology learning. |
| Biology Learning Engagement | Emotional engagement | EE1 | I feel interested when learning biology. |
| Biology Learning Engagement | Emotional engagement | EE2 | I feel a sense of enjoyment in biology learning. |
| Biology Learning Engagement | Emotional engagement | EE3 | I feel positive when I make progress in biology. |
| Biology Learning Engagement | Emotional engagement | EE4 | I feel emotionally involved in biology learning activities. |
| Continuing Biology Learning Intention | Continuing biology learning intention | CBLI1 | I intend to continue learning biology-related knowledge in the future. |
| Continuing Biology Learning Intention | Continuing biology learning intention | CBLI2 | I would like to take more biology-related courses if possible. |
| Continuing Biology Learning Intention | Continuing biology learning intention | CBLI3 | I plan to use biology knowledge in future study or life decisions. |
| Continuing Biology Learning Intention | Continuing biology learning intention | CBLI4 | I am willing to keep following biology-related scientific issues. |

Note. Items were rated from 1 = strongly disagree to 5 = strongly agree. The questionnaire was administered in Chinese; English-translated wording is provided for manuscript reporting.

**Supplementary Table S1. Full item-level descriptive statistics.**

| **Item** | **Factor** | **N** | **Mean** | **SD** | **Minimum** | **Maximum** | **Floor1 percent** | **Ceiling-5percent** | **Corrected ItemTotal r** | **Alpha if Item Deleted** |
| --- | --- | --- | --- | --- | --- | --- | --- | --- | --- | --- |
| AS1 | Autonomy support | 624 | 3.542 | 0.81 | 1 | 5 | 0.3 | 11.2 | 0.509 | 0.645 |
| AS2 | Autonomy support | 624 | 3.513 | 0.829 | 1 | 5 | 0.6 | 10.9 | 0.47 | 0.667 |
| AS3 | Autonomy support | 624 | 3.583 | 0.846 | 1 | 5 | 0.8 | 13.1 | 0.499 | 0.65 |
| AS4 | Autonomy support | 624 | 3.635 | 0.856 | 1 | 5 | 0.5 | 14.9 | 0.518 | 0.638 |
| CS1 | Competence support | 624 | 3.399 | 0.866 | 1 | 5 | 0.8 | 9.5 | 0.493 | 0.662 |
| CS2 | Competence support | 624 | 3.474 | 0.881 | 1 | 5 | 0.8 | 11.5 | 0.524 | 0.643 |
| CS3 | Competence support | 624 | 3.473 | 0.836 | 1 | 5 | 0.2 | 11.4 | 0.504 | 0.656 |
| CS4 | Competence support | 624 | 3.553 | 0.823 | 1 | 5 | 0.8 | 10.4 | 0.497 | 0.66 |
| RS1 | Relatedness support | 624 | 3.473 | 0.836 | 1 | 5 | 1 | 10.3 | 0.456 | 0.62 |
| RS2 | Relatedness support | 624 | 3.426 | 0.862 | 1 | 5 | 0.8 | 10.1 | 0.499 | 0.592 |
| RS3 | Relatedness support | 624 | 3.545 | 0.837 | 1 | 5 | 0.3 | 11.9 | 0.442 | 0.629 |
| RS4 | Relatedness support | 624 | 3.421 | 0.825 | 1 | 5 | 0.6 | 9.3 | 0.456 | 0.62 |
| AUT1 | Autonomy need satisfaction | 624 | 3.45 | 0.873 | 1 | 5 | 1 | 10.9 | 0.528 | 0.662 |
| AUT2 | Autonomy need satisfaction | 624 | 3.34 | 0.857 | 1 | 5 | 1 | 7.7 | 0.529 | 0.661 |
| AUT3 | Autonomy need satisfaction | 624 | 3.506 | 0.854 | 1 | 5 | 1.1 | 11.9 | 0.55 | 0.649 |
| AUT4 | Autonomy need satisfaction | 624 | 3.402 | 0.848 | 1 | 5 | 1 | 8.8 | 0.465 | 0.698 |
| COM1 | Competence need satisfaction | 624 | 3.417 | 0.848 | 1 | 5 | 1 | 9.9 | 0.505 | 0.67 |
| COM2 | Competence need satisfaction | 624 | 3.446 | 0.883 | 1 | 5 | 1.3 | 10.9 | 0.495 | 0.676 |
| COM3 | Competence need satisfaction | 624 | 3.388 | 0.885 | 1 | 5 | 1.6 | 9.6 | 0.513 | 0.665 |
| COM4 | Competence need satisfaction | 624 | 3.346 | 0.843 | 1 | 5 | 1.1 | 7.5 | 0.545 | 0.646 |
| REL1 | Relatedness need satisfaction | 624 | 3.381 | 0.883 | 1 | 5 | 0.8 | 9.6 | 0.539 | 0.663 |
| REL2 | Relatedness need satisfaction | 624 | 3.532 | 0.845 | 1 | 5 | 0.5 | 11.1 | 0.523 | 0.673 |
| REL3 | Relatedness need satisfaction | 624 | 3.458 | 0.869 | 1 | 5 | 1.4 | 10.4 | 0.516 | 0.677 |
| REL4 | Relatedness need satisfaction | 624 | 3.561 | 0.872 | 1 | 5 | 0.6 | 14.6 | 0.515 | 0.677 |
| BSE1 | Biology learning self-efficacy | 624 | 3.345 | 0.86 | 1 | 5 | 0.3 | 8.5 | 0.518 | 0.744 |
| BSE2 | Biology learning self-efficacy | 624 | 3.396 | 0.877 | 1 | 5 | 0.8 | 10.7 | 0.566 | 0.728 |
| BSE3 | Biology learning self-efficacy | 624 | 3.479 | 0.846 | 1 | 5 | 1.1 | 10.1 | 0.532 | 0.74 |
| BSE4 | Biology learning self-efficacy | 624 | 3.377 | 0.878 | 1 | 5 | 1.3 | 9.6 | 0.568 | 0.728 |
| BSE5 | Biology learning self-efficacy | 624 | 3.362 | 0.892 | 1 | 5 | 1.6 | 9.1 | 0.557 | 0.731 |
| SI1 | Science identity | 624 | 3.454 | 0.851 | 1 | 5 | 0.6 | 10.3 | 0.496 | 0.714 |
| SI2 | Science identity | 624 | 3.417 | 0.876 | 1 | 5 | 1.4 | 9.6 | 0.53 | 0.702 |
| SI3 | Science identity | 624 | 3.399 | 0.897 | 1 | 5 | 1.4 | 11.1 | 0.528 | 0.703 |
| SI4 | Science identity | 624 | 3.37 | 0.861 | 1 | 5 | 1.3 | 8.5 | 0.498 | 0.714 |
| SI5 | Science identity | 624 | 3.287 | 0.866 | 1 | 5 | 1.8 | 7.1 | 0.529 | 0.703 |
| BE1 | Behavioral engagement | 624 | 3.551 | 0.893 | 1 | 5 | 0.8 | 14.4 | 0.49 | 0.665 |
| BE2 | Behavioral engagement | 624 | 3.521 | 0.855 | 1 | 5 | 0.5 | 12.7 | 0.473 | 0.675 |
| BE3 | Behavioral engagement | 624 | 3.521 | 0.879 | 1 | 5 | 1.1 | 11.9 | 0.492 | 0.664 |
| BE4 | Behavioral engagement | 624 | 3.474 | 0.881 | 1 | 5 | 0.8 | 12.8 | 0.567 | 0.618 |
| CEG1 | Cognitive engagement | 624 | 3.33 | 0.867 | 1 | 5 | 1.3 | 8 | 0.476 | 0.639 |
| CEG2 | Cognitive engagement | 624 | 3.415 | 0.888 | 1 | 5 | 1.1 | 11.1 | 0.46 | 0.649 |
| CEG3 | Cognitive engagement | 624 | 3.405 | 0.893 | 1 | 5 | 1.8 | 10.1 | 0.511 | 0.617 |
| CEG4 | Cognitive engagement | 624 | 3.404 | 0.885 | 1 | 5 | 1.8 | 10.6 | 0.485 | 0.633 |
| EE1 | Emotional engagement | 624 | 3.452 | 0.864 | 1 | 5 | 0.6 | 10.6 | 0.526 | 0.649 |
| EE2 | Emotional engagement | 624 | 3.554 | 0.833 | 1 | 5 | 0.5 | 12 | 0.494 | 0.668 |
| EE3 | Emotional engagement | 624 | 3.47 | 0.844 | 1 | 5 | 0.8 | 10.6 | 0.484 | 0.674 |
| EE4 | Emotional engagement | 624 | 3.455 | 0.833 | 1 | 5 | 0.3 | 9.1 | 0.531 | 0.646 |
| CBLI1 | Continuing biology learning intention | 624 | 3.373 | 0.89 | 1 | 5 | 0.3 | 11.1 | 0.553 | 0.66 |
| CBLI2 | Continuing biology learning intention | 624 | 3.357 | 0.886 | 1 | 5 | 2.4 | 9 | 0.532 | 0.673 |
| CBLI3 | Continuing biology learning intention | 624 | 3.436 | 0.855 | 1 | 5 | 0.5 | 10.3 | 0.509 | 0.686 |
| CBLI4 | Continuing biology learning intention | 624 | 3.375 | 0.917 | 1 | 5 | 1.6 | 11.1 | 0.513 | 0.684 |

Note. N = 624. Floor 1 percent and Ceiling 5 percent indicate the percentage of responses at the lowest and highest response options.

**Supplementary Table S2. Full standardized CFA item loadings**

| **Construct** | **First OrderFactor** | **Item** | **Standardized Loading** | **SE** | **z** | **p** | **Residual Variance** | **Decision** |
| --- | --- | --- | --- | --- | --- | --- | --- | --- |
| Need-Supportive Biology Course Climate | Autonomy support | AS1 | 0.694 | 0.034 | 20.63 | <.001 | 0.518 | retained |
| Need-Supportive Biology Course Climate | Autonomy support | AS2 | 0.675 | 0.037 | 18.42 | <.001 | 0.544 | retained |
| Need-Supportive Biology Course Climate | Autonomy support | AS3 | 0.697 | 0.027 | 25.7 | <.001 | 0.515 | retained |
| Need-Supportive Biology Course Climate | Autonomy support | AS4 | 0.71 | 0.034 | 20.97 | <.001 | 0.495 | retained |
| Need-Supportive Biology Course Climate | Competence support | CS1 | 0.693 | 0.034 | 20.46 | <.001 | 0.519 | retained |
| Need-Supportive Biology Course Climate | Competence support | CS2 | 0.715 | 0.03 | 23.7 | <.001 | 0.489 | retained |
| Need-Supportive Biology Course Climate | Competence support | CS3 | 0.693 | 0.037 | 18.83 | <.001 | 0.52 | retained |
| Need-Supportive Biology Course Climate | Competence support | CS4 | 0.686 | 0.032 | 21.5 | <.001 | 0.529 | retained |
| Need-Supportive Biology Course Climate | Relatedness support | RS1 | 0.673 | 0.032 | 20.81 | <.001 | 0.548 | retained |
| Need-Supportive Biology Course Climate | Relatedness support | RS2 | 0.704 | 0.027 | 26.02 | <.001 | 0.505 | retained |
| Need-Supportive Biology Course Climate | Relatedness support | RS3 | 0.664 | 0.037 | 18.03 | <.001 | 0.559 | retained |
| Need-Supportive Biology Course Climate | Relatedness support | RS4 | 0.67 | 0.027 | 24.68 | <.001 | 0.551 | retained |
| Basic Psychological Needs | Autonomy need satisfaction | AUT1 | 0.712 | 0.036 | 19.7 | <.001 | 0.493 | retained |
| Basic Psychological Needs | Autonomy need satisfaction | AUT2 | 0.71 | 0.032 | 21.87 | <.001 | 0.497 | retained |
| Basic Psychological Needs | Autonomy need satisfaction | AUT3 | 0.721 | 0.034 | 21.38 | <.001 | 0.48 | retained |
| Basic Psychological Needs | Autonomy need satisfaction | AUT4 | 0.669 | 0.031 | 21.82 | <.001 | 0.552 | retained |
| Basic Psychological Needs | Competence need satisfaction | COM1 | 0.692 | 0.031 | 22.2 | <.001 | 0.522 | retained |
| Basic Psychological Needs | Competence need satisfaction | COM2 | 0.694 | 0.032 | 21.45 | <.001 | 0.518 | retained |
| Basic Psychological Needs | Competence need satisfaction | COM3 | 0.705 | 0.035 | 20.23 | <.001 | 0.503 | retained |
| Basic Psychological Needs | Competence need satisfaction | COM4 | 0.715 | 0.029 | 24.87 | <.001 | 0.489 | retained |
| Basic Psychological Needs | Relatedness need satisfaction | REL1 | 0.718 | 0.028 | 25.31 | <.001 | 0.485 | retained |
| Basic Psychological Needs | Relatedness need satisfaction | REL2 | 0.701 | 0.029 | 24.38 | <.001 | 0.509 | retained |
| Basic Psychological Needs | Relatedness need satisfaction | REL3 | 0.702 | 0.033 | 21.25 | <.001 | 0.508 | retained |
| Basic Psychological Needs | Relatedness need satisfaction | REL4 | 0.702 | 0.037 | 19.17 | <.001 | 0.508 | retained |
| Biology Learning Self-Efficacy | Biology learning self-efficacy | BSE1 | 0.667 | 0.03 | 22.42 | <.001 | 0.555 | retained |
| Biology Learning Self-Efficacy | Biology learning self-efficacy | BSE2 | 0.7 | 0.035 | 19.74 | <.001 | 0.51 | retained |
| Biology Learning Self-Efficacy | Biology learning self-efficacy | BSE3 | 0.673 | 0.032 | 20.76 | <.001 | 0.547 | retained |
| Biology Learning Self-Efficacy | Biology learning self-efficacy | BSE4 | 0.702 | 0.033 | 21.09 | <.001 | 0.508 | retained |
| Biology Learning Self-Efficacy | Biology learning self-efficacy | BSE5 | 0.698 | 0.029 | 24.41 | <.001 | 0.513 | retained |
| Science Identity | Science identity | SI1 | 0.655 | 0.032 | 20.41 | <.001 | 0.572 | retained |
| Science Identity | Science identity | SI2 | 0.681 | 0.028 | 24.66 | <.001 | 0.536 | retained |
| Science Identity | Science identity | SI3 | 0.684 | 0.028 | 24.09 | <.001 | 0.533 | retained |
| Science Identity | Science identity | SI4 | 0.658 | 0.036 | 18.19 | <.001 | 0.567 | retained |
| Science Identity | Science identity | SI5 | 0.679 | 0.035 | 19.35 | <.001 | 0.539 | retained |
| Biology Learning Engagement | Behavioral engagement | BE1 | 0.691 | 0.032 | 21.42 | <.001 | 0.522 | retained |
| Biology Learning Engagement | Behavioral engagement | BE2 | 0.673 | 0.03 | 22.31 | <.001 | 0.547 | retained |
| Biology Learning Engagement | Behavioral engagement | BE3 | 0.69 | 0.036 | 19.13 | <.001 | 0.524 | retained |
| Biology Learning Engagement | Behavioral engagement | BE4 | 0.733 | 0.03 | 24.34 | <.001 | 0.462 | retained |
| Biology Learning Engagement | Cognitive engagement | CEG1 | 0.679 | 0.034 | 19.71 | <.001 | 0.539 | retained |
| Biology Learning Engagement | Cognitive engagement | CEG2 | 0.674 | 0.028 | 24.38 | <.001 | 0.546 | retained |
| Biology Learning Engagement | Cognitive engagement | CEG3 | 0.705 | 0.036 | 19.47 | <.001 | 0.502 | retained |
| Biology Learning Engagement | Cognitive engagement | CEG4 | 0.689 | 0.031 | 22.51 | <.001 | 0.526 | retained |
| Biology Learning Engagement | Emotional engagement | EE1 | 0.713 | 0.032 | 22.31 | <.001 | 0.492 | retained |
| Biology Learning Engagement | Emotional engagement | EE2 | 0.688 | 0.031 | 21.92 | <.001 | 0.527 | retained |
| Biology Learning Engagement | Emotional engagement | EE3 | 0.684 | 0.035 | 19.6 | <.001 | 0.532 | retained |
| Biology Learning Engagement | Emotional engagement | EE4 | 0.71 | 0.03 | 23.6 | <.001 | 0.496 | retained |
| Continuing Biology Learning Intention | Continuing biology learning intention | CBLI1 | 0.723 | 0.034 | 21.19 | <.001 | 0.477 | retained |
| Continuing Biology Learning Intention | Continuing biology learning intention | CBLI2 | 0.71 | 0.033 | 21.58 | <.001 | 0.496 | retained |
| Continuing Biology Learning Intention | Continuing biology learning intention | CBLI3 | 0.69 | 0.03 | 22.63 | <.001 | 0.524 | retained |
| Continuing Biology Learning Intention | Continuing biology learning intention | CBLI4 | 0.705 | 0.033 | 21.54 | <.001 | 0.503 | retained |

Note. CFA = confirmatory factor analysis. All items were retained.

**Supplementary Table S3. Group mean data for Figure 3.**

| **Institution_Type** | **n** | **Construct** | **Mean** | **SD** | **SE** |
| --- | --- | --- | --- | --- | --- |
| comprehensive university | 247 | Need-Supportive Biology Course Climate | 3.514 | 0.518 | 0.033 |
| comprehensive university | 247 | Basic Psychological Needs | 3.462 | 0.511 | 0.033 |
| comprehensive university | 247 | Biology Learning Self-Efficacy | 3.365 | 0.616 | 0.039 |
| comprehensive university | 247 | Science Identity | 3.415 | 0.623 | 0.04 |
| comprehensive university | 247 | Biology Learning Engagement | 3.461 | 0.511 | 0.032 |
| comprehensive university | 247 | Continuing Biology Learning Intention | 3.399 | 0.659 | 0.042 |
| teacher education university | 118 | Need-Supportive Biology Course Climate | 3.518 | 0.464 | 0.043 |
| teacher education university | 118 | Basic Psychological Needs | 3.387 | 0.529 | 0.049 |
| teacher education university | 118 | Biology Learning Self-Efficacy | 3.441 | 0.615 | 0.057 |
| teacher education university | 118 | Science Identity | 3.286 | 0.524 | 0.048 |
| teacher education university | 118 | Biology Learning Engagement | 3.421 | 0.528 | 0.049 |
| teacher education university | 118 | Continuing Biology Learning Intention | 3.36 | 0.649 | 0.06 |
| applied undergraduate university | 114 | Need-Supportive Biology Course Climate | 3.433 | 0.553 | 0.052 |
| applied undergraduate university | 114 | Basic Psychological Needs | 3.363 | 0.542 | 0.051 |
| applied undergraduate university | 114 | Biology Learning Self-Efficacy | 3.305 | 0.651 | 0.061 |
| applied undergraduate university | 114 | Science Identity | 3.263 | 0.652 | 0.061 |
| applied undergraduate university | 114 | Biology Learning Engagement | 3.424 | 0.524 | 0.049 |
| applied undergraduate university | 114 | Continuing Biology Learning Intention | 3.329 | 0.73 | 0.068 |
| agricultural/life-science university | 145 | Need-Supportive Biology Course Climate | 3.527 | 0.52 | 0.043 |
| agricultural/life-science university | 145 | Basic Psychological Needs | 3.488 | 0.556 | 0.046 |
| agricultural/life-science university | 145 | Biology Learning Self-Efficacy | 3.465 | 0.654 | 0.054 |
| agricultural/life-science university | 145 | Science Identity | 3.512 | 0.62 | 0.052 |
| agricultural/life-science university | 145 | Biology Learning Engagement | 3.53 | 0.531 | 0.044 |
| agricultural/life-science university | 145 | Continuing Biology Learning Intention | 3.428 | 0.626 | 0.052 |

Note. These values provide the source data for Figure 2.

**Supplementary Table S4. Selected group-specific structural path estimates by institution type.**

| **Institution type** | **n** | **Path** | **β** | **SE** | **p** | **95% CI** |
| --- | --- | --- | --- | --- | --- | --- |
| Comprehensive university | 247 | Course Climate → Basic Needs | 0.479 | 0.056 | < .001 | [0.369, 0.590] |
| Comprehensive university | 247 | Basic Needs → Learning Engagement | 0.148 | 0.061 | .015 | [0.028, 0.267] |
| Comprehensive university | 247 | Science Identity → Learning Engagement | 0.224 | 0.057 | < .001 | [0.111, 0.337] |
| Comprehensive university | 247 | Self-Efficacy → Learning Engagement | 0.226 | 0.056 | < .001 | [0.115, 0.337] |
| Comprehensive university | 247 | Learning Engagement → Continuing Intention | 0.306 | 0.068 | < .001 | [0.171, 0.440] |
| Teacher education university | 118 | Course Climate → Basic Needs | 0.556 | 0.077 | < .001 | [0.403, 0.709] |
| Teacher education university | 118 | Basic Needs → Learning Engagement | 0.523 | 0.092 | < .001 | [0.340, 0.706] |
| Teacher education university | 118 | Science Identity → Learning Engagement | 0.004 | 0.076 | .959 | [-0.146, 0.154] |
| Teacher education university | 118 | Self-Efficacy → Learning Engagement | 0.196 | 0.083 | .020 | [0.032, 0.360] |
| Teacher education university | 118 | Learning Engagement → Continuing Intention | 0.202 | 0.100 | .046 | [0.003, 0.401] |
| Applied undergraduate university | 114 | Course Climate → Basic Needs | 0.597 | 0.076 | < .001 | [0.447, 0.747] |
| Applied undergraduate university | 114 | Basic Needs → Learning Engagement | 0.315 | 0.097 | .002 | [0.123, 0.508] |
| Applied undergraduate university | 114 | Science Identity → Learning Engagement | 0.343 | 0.088 | < .001 | [0.169, 0.518] |
| Applied undergraduate university | 114 | Self-Efficacy → Learning Engagement | 0.108 | 0.078 | .168 | [-0.046, 0.263] |
| Applied undergraduate university | 114 | Learning Engagement → Continuing Intention | 0.336 | 0.092 | < .001 | [0.154, 0.518] |
| Agricultural/life-science university | 145 | Course Climate → Basic Needs | 0.630 | 0.065 | < .001 | [0.501, 0.758] |
| Agricultural/life-science university | 145 | Basic Needs → Learning Engagement | 0.284 | 0.088 | .002 | [0.110, 0.457] |
| Agricultural/life-science university | 145 | Science Identity → Learning Engagement | 0.360 | 0.075 | < .001 | [0.211, 0.509] |
| Agricultural/life-science university | 145 | Self-Efficacy → Learning Engagement | 0.127 | 0.077 | .100 | [-0.025, 0.280] |
| Agricultural/life-science university | 145 | Learning Engagement → Continuing Intention | 0.150 | 0.091 | .102 | [-0.030, 0.330] |

Note. Estimates are standardized coefficients. Because the group sizes were unequal, group-specific estimates were treated as exploratory. Smaller groups may have lower statistical power and less precise parameter estimates; therefore, differences in statistical significance across groups should not be interpreted by themselves as evidence of significant between-group differences in structural paths.
